# Spatio-temporal variability of mesozooplankton distribution along the Canary Current Large Marine Ecosystem: a regional perspective

**DOI:** 10.1093/plankt/fbae079

**Published:** 2025-01-30

**Authors:** Yassine Goliat, Omar Ettahiri, Tarik Baibai, Nadia Rharbi, Stamatina Isari

**Affiliations:** Laboratory of Health and Environment, Department of Biology, Faculty of Sciences Aïn-Chock, Hassan II University, Km 8 Roure d'El Jadida, B.P. 5366, Casablanca 20100, Morocco; Laboratory of Marine Plankton Ecology, Department of Oceanography, National Institute of Fisheries Research (INRH), 2 Boulevard Sidi Abderrahmane, Casablanca 20250, Morocco; Laboratory of Marine Plankton Ecology, Department of Oceanography, National Institute of Fisheries Research (INRH), 2 Boulevard Sidi Abderrahmane, Casablanca 20250, Morocco; Laboratory of Marine Plankton Ecology, Department of Oceanography, National Institute of Fisheries Research (INRH), 2 Boulevard Sidi Abderrahmane, Casablanca 20250, Morocco; Laboratory of Health and Environment, Department of Biology, Faculty of Sciences Aïn-Chock, Hassan II University, Km 8 Roure d'El Jadida, B.P. 5366, Casablanca 20100, Morocco; Institute of Marine Research (IMR), PO Box 1870, Nordnes, Bergen NO-5817, Norway

**Keywords:** mesozooplankton distribution, assemblage structure, copepod community, Canary Current Large Marine Ecosystem, northwest Africa, coastal upwelling, eastern boundary large marine ecosystems

## Abstract

The Canary Current Large Marine Ecosystem (CCLME), extending from Cape Spartel in Morocco to Guinea-Bissau, supports high primary and fisheries productivity driven by permanent or seasonal upwelling activity. During the current study, mesozooplankton and hydrographic sampling were conducted across the CCLME in the spring/summer of 2017 and the autumn/winter of 2019. The total mesozooplankton abundance and dry weight were found to be higher in 2017, partly due to the summer reproduction cycle of diplostracans. A prominent latitudinal gradient was observed in both the mesozooplankton standing stock and assemblage structure closely linked to a significant shift in oceanographic regimes at Cape Blanc (21^°^N). The area south of Cape Blanc, sampled during the upwelling relaxation in both years, was occupied by warmer South Atlantic Central Waters showing elevated mesozooplankton stock with a tropical assemblage structure. In contrast, cooler and more saline waters north of Cape Blanc, a result of the upwelling regime in that area, explained part of the observed variation in mesozooplankton composition among subregions and sampling periods. Our findings indicate that aside from the upwelling activity, spatiotemporal variation of mesoscale processes and topographical features at a subregional level may also shape mesozooplankton stock and assemblage structure in the CCLME.

## INTRODUCTION

The Canary Current Large Marine Ecosystem (CCLME) stretches from Cape Spartel at the northern of Morocco (36°N) to southern Guinea Bissau (8°N) and is one of the four Eastern Boundary Upwelling Systems (EBUS) in the globe (Chavez and Messié, 2009; Fréon *et al.*, 2009; Kämpf and Chapman, 2016), ranking third in terms of primary productivity and exhibiting the highest level of fisheries production among all African Upwelling Large Marine Ecosystems (Déniz-González *et al.*, 2016). CCLME features two distinct water masses: the North Atlantic Central Water (NACW), flowing equatorward via the Canary Current, and the South Atlantic Central Water (SACW), which flows poleward through the Mauritanian Current. These water masses are separated by a dynamic front at 21°N (Cape Blanc), undergoing significant mixing and interlacing (Arístegui *et al.*, 2006, 2009). The high regional productivity is primarily supported by the strength of north-easterly trade winds that blow towards the equator, inducing the offshore movement of surface water and leading to an inshore upwelled current rich in nutrients (Kämpf and Chapman, 2016). Due to a remarkable seasonality in the alongshore trade wind forcing, upwelling regime intensity can vary spatiotemporally along the CCLME, dividing the ecosystem into distinct subregions (e.g. Arístegui *et al.*, 2009; Cropper *et al.*, 2014; Benazzouz *et al.*, 2014a). Cape Blanc at the borders of Mauritania (21°N), has been regarded as an important topographical limit where major changes in upwelling dynamics occur. Southern of Cape Blanc, the upwelling is only seasonal and occurs during winter/spring (e.g. Arístegui *et al.*, 2009; Cropper *et al.*, 2014). On the other hand, a year-round strong upwelling characterizes the central CCLME northern of Cape Blanc (21°N–26°N), while further northern (26°N–33°N) the upwelling though remains permanent, is weaker, intensifying during summer. Nevertheless, upwelling activity in the northern part of the African CCLME (33°N–36°N) is characterized by a weak to absent upwelling activity.

In addition to the upwelling regime, the productivity of the Northwest African coast is closely linked to a high degree of mesoscale oceanographic variability and the topographical complexity of the region (e.g. proximity to the islands, presence of capes) (Barton *et al.*, 1998). The presence of mesoscale structures is particularly evidenced in the area of the Canary Eddy Corridor (22°N–29°N) (Sangrà *et al.*, 2009) around the Canary Islands, but also on the Moroccan coast with the presence of upwelling filaments in proximity to Cape Ghir (31°N) (Salah *et al.*, 2012; Sangrà *et al.*, 2015) and Cape Juby (27^o^N) (Marcello *et al.*, 2011). Local spots of productivity with high importance as feeding and nursery grounds for small pelagic fish have also been highlighted in other areas across CCLME, both north and south of Cape Blanc (e.g. Guénette *et al.*, 2014; Brochier *et al.*, 2018; Tiedemann *et al.*, 2018).

Mesozooplankton play a critical role in both ecological and biogeochemical processes, as they channel energy from the lower trophic levels to higher ones and impact biochemical cycling (Batten *et al.*, 2016; Steinberg and Landry, 2017). Our knowledge regarding the ecological aspects of this important food web link in the CCLME region still lags behind that of other EBUS (e.g. Verheye *et al.*, 2016; Venegas *et al.*, 2024). Although, the first mesozooplankton studies within CCLME date back almost a century ago (Berraho *et al.*, 2015), we still lack a comprehensive understanding regarding the dynamics of mesozooplankton on a regional level. As summarized by (Hernández-León *et al.*, 2007; Berraho *et al.*, 2015), the majority of mesozooplankton research conducted in the CCLME has predominantly focused on regions situated north of Cape Blanc (21°N), with a special emphasis on the Canary Island archipelago. Studies in this area have primarily explored the effects of upwelling filaments on the ecophysiological traits of mesozooplankton and subsequent implications for vertical fluxes (Hernández-León *et al.*, 2007; Berraho *et al.*, 2015), while just recently, size spectra (Couret *et al.*, 2023a) and modeling approaches (Couret *et al.*, 2023b), have been incorporated to address mesozooplankton spatiotemporal dynamics.

Outside the Canary Islands basin, mesozooplankton distribution patterns (particularly those of copepods) in relation to hydrography, have been mostly examined at highly localized spatial scales (Kuipers *et al.*, 1993; Salah *et al.*, 2012; Zaafa *et al.*, 2014; Berraho *et al.*, 2019a; Berraho *et al.*, 2019b). Broader scale published data are limited, and mostly stemming from mesozooplankton sampling conducted several decades ago. Such studies were conducted in the framework of German research efforts during the 70s (Postel *et al.,* 1995), but also of Russian research voyages (since 1994) both along the Moroccan coastal waters (i.e, Somoue *et al.*, 2005; Lidvanov *et al.*, 2018) and south of Cape Blanc (Sirota *et al.*, 2004; Glushko and Lidvanov, 2012; Lidvanov *et al.*, 2022). Notably, information from the latter research expeditions is predominantly accessible through Russian scientific literature (e.g. Glushko and Lidvanov, 2012; Shukhgalter and Lidvanov, 2018; Lidvanov *et al.*, 2022). We are still lacking regional mesozooplankton studies covering the entire CCLME, that would allow spatiotemporal comparisons and future necessary modeling efforts of zooplankton communities in view of climate change (Ratnarajah *et al.*, 2023). The latter requires not only intensive sampling effort but also standardized sampling design and protocols.

Here, we examine the broadscale distribution patterns of mesozooplankton assemblages along the coast of North-West Africa encompassing sub-regions of different upwelling regimes across the CCLME. Mesozooplankton and hydrographic sampling for the current study took place in two distinct years representing different seasons (2017: spring/summer, 2019: autumn/winter) in the framework of the EAF-Nansen programme of the Food and Agriculture Organization of the United Nations (FAO). Our main aim was to trace seasonal/interannual and subregional variability in mesozooplankton distribution patterns, in association with the environmental conditions. We hypothesized that mesozooplankton abundance and dry weight patterns in the area as well as assemblage structure will largely reflect the hydrographic conditions imposed by the upwelling conditions and topography of each region. We expect that our findings will enable a better understanding of the broader ecological patterns at a regional level and provide a valuable data source for future work in the CCLME, allowing for tracing the climate change-driven shifts in distribution patterns and changes in abundance, and the potential consequences for higher trophic levels (e.g. small pelagic fish).

## METHOD

### Sampling design

**Fig. 1:**
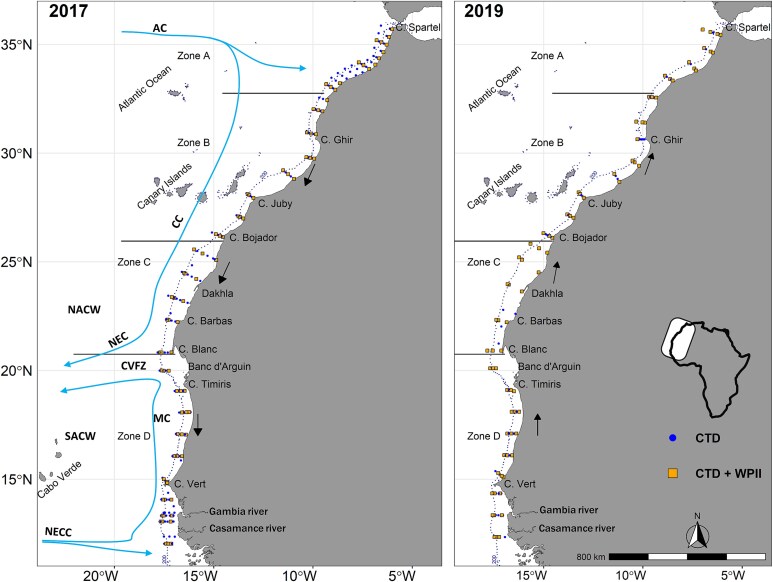
Maps of the study area in 2017 and 2019. Stations of hydrographic (CTD) and mesozooplankton (WPII) sampling are shown. Sampling directions are indicated with arrows. Major water mass circulation and oceanographic features adapted from Arístegui *et al.*, (2009) are indicated (AC: Azores Current, CC: Canary Current, CVFZ: Cape Verde Frontal Zone, MC: Mauritanian Current, NEC: North Equatorial Current, NECC: North Equatorial Countercurrent, NACW: North Atlantic Central Water, SACW: South Atlantic Central Water). The Zones of distinct upwelling regime shown in the study area are based on Arístegui *et al.*, (2009) and Cropper *et al.*, (2014) [i.e. Zone A: 36°N—33°N, weak to absent upwelling; Zone B: 33°N -26°N, permanent weak upwelling; Zone C: 26°N -21°N, permanent strong upwelling; Zone D: 21°N—12°N, seasonal upwelling]. The position of the isobath 200 m is indicated with a dashed line.

The study area covered the Northwest African coast (NWA), between 36°N north of Morocco and 12°N north of Guinea-Bissau (Fig. 1). Sampling was done on board the R/V *Dr Fridtjof Nansen* during seven surveys conducted in spring/summer 2017 and autumn/winter 2019 (Table S1). Mesozooplankton was sampled at 158 stations positioned across transects running perpendicular to the coast (2017: 82 stations, 2019: 76 stations, Table S1). Overall, stations were located at three isobathic strata (Str) at depths of 30 m (Str1), 100 m (Str2) and 500 m (Str3, including also few sites positioned at depths greater than 500 m). Tows were performed using a WP2 net (56 cm diameter, area of 0.25 m^2^ and 180 μm mesh size), equipped with a Hydrobios (438 115) mechanical flowmeter and hauled vertically at each station at a speed of ~ 0.5 m. s^−1^. Sampling was conducted during day or night (Fig. S1), from the depth of 200 m to the surface for stations with a bottom depth exceeding 200 m (or from 3 to 5 m above the sea floor at shallower stations). Str1,2 were not expected to be influenced by sampling time, as the tows covered their entire water column. In Str3 (stations ≥500 m depth, sampled at 0–200 m), mesozooplankton diel vertical migration (DVM) could have an effect and this was further explored (see data analysis).

A total of 332 Conductivity Temperature Depth (CTD) casts were done in both years across the studied area, covering both coastal and offshore waters (2017: 200 casts; 2019: 132 casts, Table S1). In 2017, hydrographic sampling was conducted using a Seabird 911 CTD, equipped with a Chelsea Mk III Aquatracka fluorometer and an SBE 43 oxygen sensor. In 2019, a Sea-Bird 911plus CTD profiler was deployed, equipped with a WET Labs ECO-AFL fluorometer for *in situ* fluorescence measurement. The CTD sensors aboard R/V *Dr Fridtjof Nansen* undergo routine pre- and post-use calibration by the manufacturer every 1–2 years. During each survey, sensor data are rigorously validated. Fluorescence was employed to estimate Chlorophyll-a (Chl-a) concentrations, with fluorometer data validated against onboard Chl-a measurements. These measurements were obtained from water samples collected at various depths using Niskin bottles. The Chl-a assay involved extraction with 90% acetone, followed by centrifugation, and analysis using a fluorometer (model 10 AU, Turner Designs Inc., Sunnyvale, CA, USA) in accordance with Welschmeyer (1994) and Jeffrey and Humphrey (1975). Salinity measurements were cross-verified onboard using a Portasal Salinometer (Model 8410A), while oxygen measurements were validated using Winkler titration, following the protocol outlined by Grasshoff *et al.* (1983).

### Sample processing and analysis

Each mesozooplankton sample was halved on board using a Motoda box. The first half was dried on pre-weighed aluminum trays for 24 h at ~ 60°C, for the estimation of the mesozooplankton dry weight as a biomass measure. The second half was directly fixed with a 4% borax-buffered formaldehyde solution and was used for further estimation of the total abundance and species composition. Seven samples were not served for taxonomic analysis due to their poor preservation (one sample in 2017 and six samples in 2019).

Taxonomic identification was done to the lowest possible taxonomic level based on morphological characteristics using available sources (Rose, 1933; Trégouboff and Rose, 1957; Wiafe and Frid, 2001; Richardson *et al.*, 2013; Razouls *et al.*, 2024). In this study, we use the term Paracalanus parvus^*^ to refer to the *P. parvus* species complex (Kasapidis *et al.*, 2018). Abundance and dry weight values were expressed as individuals per square meter (ind. m^−2^) and grams per square meter (g m^−2^) to provide information on the total mesozooplankton standing stock in the first 200 m of the water column. However, to allow comparison with other studies in the area we also provide, as supplementary material, density expressed as ind. m^−3^.

### Data analyses

Due to the high variability of the environmental parameters across the study area, we followed an “a priori” division of four latitudinal Zones (Fig. 1A–D) based on previous knowledge on the hydrological features and upwelling dynamics within the CCLME (e.g. Arístegui *et al.*, 2009; Cropper *et al.*, 2014; Benazzouz *et al.*, 2014a). The Zones were defined as follows: Zone A (36°N–33°N) with weak to absent upwelling activity, Zone B (33°N–26°N) with weak and permanent upwelling activity, stronger in summer and autumn, Zone C (26°N–21°N) with strong and permanent upwelling activity throughout the year, peaking from spring to autumn and Zone D (21°N–12°N) with seasonal upwelling, primarily occurring in winter.

Exploration for day-night sampling effects in St3 (stations ≥500 m depth, sampled at 0–200 m) is provided in Supplement S2. Comparisons within Zones of balanced day/night sampling in Str3 (assuming consistent zooplankton behavior among nearby stations), showed no significant day vs. night differences in mesozooplankton stock and assemblage structure. Latitudinal trends in zooplankton stock were also found similar, regardless of the inclusion of the deeper stratum, and the clustering in Str 3 mirrored the latitudinal separation of shallower stations (Str1, 2). Therefore, for the purpose of this work, we have included Str3 (25% of the sampling grid) in the same analysis as the other two strata.

Contour maps of surface temperature, salinity, Chl-a and oxygen were made using the Ocean Data View and Data-Interpolating Variational Analysis (ODV version 5.7.0, Schlitzer, 2024). All other plotting and analyses were performed in the R programming language (version 4.3.3, R Core Team, 2024) and the R studio software (version 2024.04.0, RStudio Team, 2024). Plotting was done using the packages “ggOceanMaps” (version 2.0.0, Vihtakari, 2023) and “ggplot2” (version 3.4.2, Wickham, 2016). Statistics were performed using the packages “stats and vegan” (version 2.6-4, Oksanen *et al.*, 2022).

Significant differences in the surface hydrological parameters were tested across Zones and strata in each year by Permutational Multivariate Analysis of Variance (PERMANOVA, Anderson, 2001, 2017) with 999 permutations implemented, on the Euclidean distance matrix of prior normalized data, via the *adonis2* function from the “vegan” package. Pairwise tests were made with the function *pairwise.adonis2* (Martinez Arbizu, 2020). Seasonal/interannual and latitudinal (Zones A-D) differences in total mesozooplankton abundance and dry weight were tested by one-way ANOVA on log-transformed data, following confirmation of normality (Shapiro–Wilk test, α = 0.05) and homogeneity of variance (Levene’s test, α = 0.05). The non-parametric Kruskal-Wallis H-test and *post hoc* Dunn’s test were used for comparisons of univariate variables among the latitudinal Zones (A-D).

Hierarchical clustering and non-metric multidimensional scaling (nMDS) analysis (Clarke *et al.*, 2014), were used to investigate the copepod and diplostracan assemblage structure. To account for the influence of rare taxa and minimize the impact of dominating taxa, data was square-root transformed and a dissimilarity matrix was built using the Bray–Curtis dissimilarity index. Clustering was performed on the dissimilarity matrix using group-average linkage. Taxa with a relative density > 3% in at least one station were further clustered by calculating the Bray–Curtis dissimilarity index on pairs of taxa based on standardized abundances. To identify the taxa contributing to the 70% similarity of the clustered samples we performed Similarity Percentage analysis (SIMPER) using the *simper* function of the “vegan” package. This package was also used to calculate the diversity indices (taxonomic richness and Shannon-Weiner (H, Shannon and Weaver, 1949) based on the copepod and diplostracan genera encountered in the samples. PERMANOVA routine also tested for differences in copepod and diplostracan assemblages for each year (Zones, strata) based on the Bray–Curtis dissimilarity matrix used for clustering.

To relate the assemblage structure with the environment, selected environmental vectors were fitted on the nMDS ordination plot using the *envfit* function of the “vegan” package in R (Oksanen *et al.*, 2022). This analysis was performed separately for each year, as well as for both years combined, to identify common trends. The function *envfit* uses a permutation process to identify which environmental vectors most accurately determine the distribution of assemblage structure. The ordination scores produced by the nMDS are related by regression analysis with the selected environmental variables. The environmental variables are the dependent variables that are explained by the ordination scores, and each dependent variable is analyzed separately. Significance is tested by permutation tests (999 permutations) and *p*-values were Bonferroni-corrected. The parameters selected during our study were station sampling depth and averages of temperature, salinity, Chl-a and oxygen at the 0–30 m layer (adjusting for the shallower stations in Str1).

## RESULTS

### Environmental conditions

**Fig. 2:**
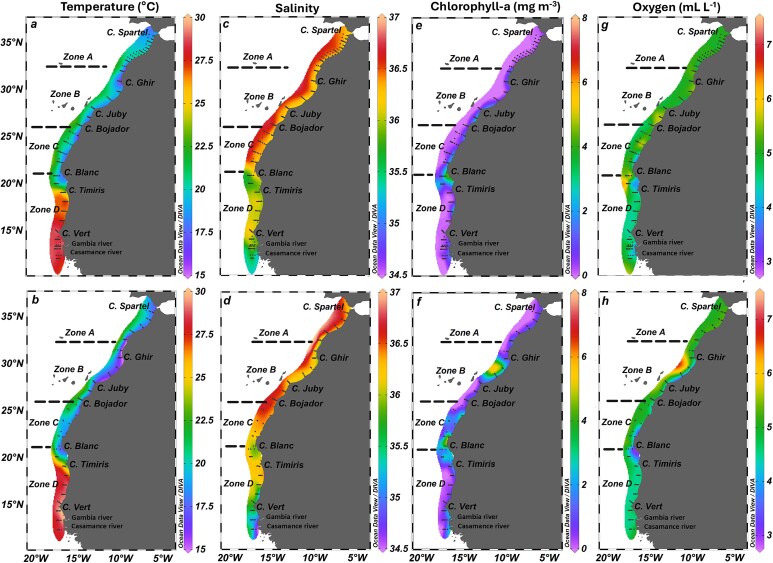
Maps of horizontal distribution of temperature (°C), salinity, Chlorophyll-a (mg m^−3^) and oxygen (mL L^−1^) along the studied area in 2017 (**a**, **c**, **e**, **g**) and 2019 (**b**, **d**, **f**, **h**). Upwelling Zones (A, B, C, D) are shown. Contours are based on 200 CTD stations in 2017 (A: 42, B: 43, C:39, D: 76) and 132 in 2019 (A: 21, B: 43, C:19, D: 49).

**Fig. 3:**
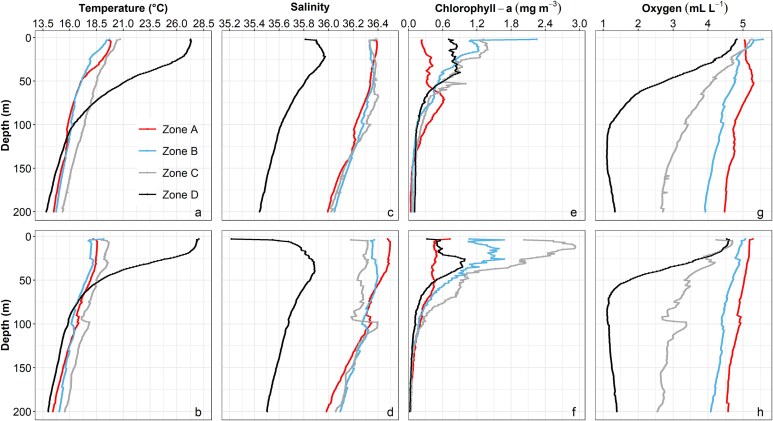
Mean vertical profiles of temperature, salinity, Chl-a and oxygen in the upper 200 m layer of each upwelling Zone (A, B, C, D), in 2017 (**a**, **c**, **e**, **g**) and 2019 (**b**, **d**, **f**, **h**). Profiles are based on 200 CTD stations in 2017 (A: 42, B: 43, C:39, D: 76) and 132 in 2019 (A: 21, B: 43, C:19, D: 49).

Contour maps of temperature, salinity, Chl-a and oxygen levels at 10 m depth (Fig. 2) and their mean depth profiles in the four latitudinal Zones (Fig. 3) showed a strong spatial variability across the CCLME. PERMANOVA analysis in each year revealed statistically significant differences across Zones and strata and an important interaction (Table I). Pairwise differences are shown in Tables S2 and S3.

**Table I:** PERMANOVA analysis results testing the effect of the latitudinal zones and the sampling strata on the hydrological parameters at 10 m (two-way PERMANOVAs) and on the copepod and diplostracan (one-way PERMANOVAs) in 2017 and 2019

		**Df**	**Sum Of Sqs**	**R** ^**2**^	**F**	**Pr (>F)**
**Hydrology**						
**2017**	**Zone**	3	336.280	0.422	52.055	0.001^***^
	**Stratum**	2	30.140	0.038	6.999	0.001^***^
	Zone^*^Stratum	6	24.760	0.031	1.916	0.017^*^
	**Residual**	188	404.820	0.509		
**2019**	**Zone**	3	247.320	0.472	45.850	0.001^***^
	**Stratum**	2	28.020	0.053	7.792	0.001^***^
	Zone^*^Stratum	6	32.900	0.063	3.049	0.001^***^
	**Residual**	120	215.760	0.412		
**Zooplankton**						
**2017**	**Zone**	3	2.382	0.211	6.879	0.001^***^
	**Residual**	77	8.886	0.789		
	**Total**	80	11.268	1		
	**Stratum**	1	1.474	0.131	11.892	0.001^***^
	**Residual**	79	9.794	0.869		
	**Total**	80	11.268	1		
**2019**	**Zone**	3	3.616	0.324	10.523	0.001^***^
	**Residual**	66	7.561	0.676		
	**Total**	69	11.177	1		
	**Stratum**	1	0.802	0.072	5.259	0.001^***^
	**Residual**	68	10.375	0.928		
	**Total**	69	11.1771	1		

In both years, surface temperature showed a wide range, with a clear latitudinal shift around 21°N (Fig. 2a and b). The upper 50 m in Zones A-C experienced warmer and more stratified conditions in spring/summer 2017 compared to early winter 2019 (Fig. 3a and b). Cooler, less saline and Chl-a-rich waters dominated the nearshore region from Cape Spartel (36°N) to Cape Blanc (21°N), indicating coastal upwelling. Interestingly, elevated Chl-a and oxygen levels were detected between Cape Juby (28°N) and Cape Ghir (31°N) in 2019 (Fig. 2f). Significant differences among all Zones North of Cape Banc were found in 2019, with a stronger distinction of the inshore area (Str1) in 2017 (Table S3).

South of Cape Blanc (Zone D), higher surface water temperatures (Fig. 2a and b) and a well-stratified water column (Fig. 3a and b) with a gradual reduction of oxygen down to 50 m were observed in both years. Less saline and rich in Chl-a waters were predominantly found near Cape Blanc and south of Cape Vert (discharge of Gambia River and Casamance River, see Fig. 2), with this trend becoming more pronounced in 2019 (Figs 2c-d and 3c-d). Notably, low oxygen waters were observed in both years around Cape Blanc (i.e. frontal Zone). Differences between sampling strata were not significant South of Cape Blanc (Table S3).

### Mesozooplankton total abundance and dry weight

**Fig. 4:**
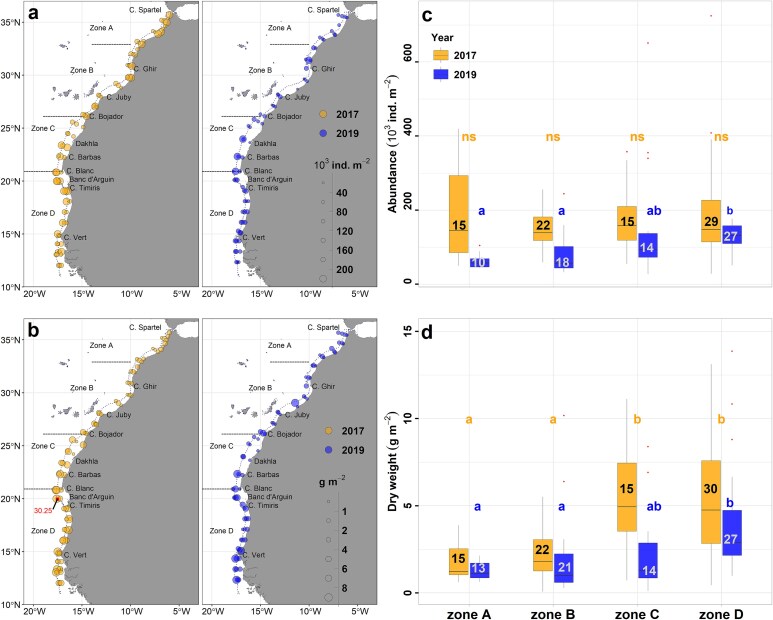
Distribution maps of (**a**) total abundance and (**b**) total dry weight in 2017 (maximum of 30.25 g m^−2^ is indicated on the map) and 2019. Box plots of (**c**) total abundance and (**d**) total dry weight in the four upwelling Zones (A, B, C, and D) in 2017 and 2019. The size of the box plot is determined by the upper and lower quartiles, with the median indicated by a horizontal line within each box. Outliers are represented by dots outside the boxes. Dunn’s *post hoc* test output is displayed as letters above the box plots (the difference in letters signifies a statistically significant difference within each years). The number of stations of each zone is shown inside the boxes.

Figure 4 shows the horizontal distribution of mesozooplankton stock. Both abundance and dry weight values varied largely across the CCLME (Fig. 4a and b; Table S4), with significantly higher mean values in 2017 (abundance: *F* = 28.07, df = 1, *P <* 0.001; dry weight: *F* = 11.06, df = 1, *P* < 0.01). Zones comparison in each year are shown in Fig. 4c and d. Mean abundance among Zones differed significantly only in 2019 (2017: *H* = 0.495, df = 3, *P* = 0.92; 2019: *H* = 20.218, df = 3, *P* < 0.001), with Zone D having higher values than A and B (Fig. 4b). For dry weight, significant differences were observed in both years (2017: *H* = 27.65, df = 3, *P* < 0.001; 2019: *H* = 19.11, df = 3, *P* < 0.001). In 2017, Zones C and D had higher values than A and B, whereas in 2019 only Zone D differed significantly from A and B (Fig. 4c and d).

### Taxonomic composition, assemblage structure and relation with the environment

In total, 25 mesozooplankton groups (Table II) and 152 copepod taxa were identified (Table III; 2017: 104, 2019: 134) in the samples. Copepods dominated both years, with a relative density of over 80%, while diplostracans, appendicularians, chaetognaths, doliolids and pteropods followed in rank order (Table II). Distribution maps for the major groups are presented in Fig. S2. The diplostracan taxa *Penilia avirostris* and *Podon* spp. contributed more in 2017 (Table II), with the latter mainly distributed north of Cape Blanc (Fig. S2).

**Table II:** Mean abundance (ind. m^−2^) and mean relative contribution (%) of mesozooplankton groups identified in the samples collected in 2017 (N = 81) and 2019 (N = 70).

	**Mean Abundance**		**Mean relative contribution $ \boldsymbol{(\%)} $**	
**Taxa**	**2017**	**2019**	**2017**	**2019**
**Holoplankton**				
**Copepods**	145 229.5 (10913.5)	92 246 (9272.8)	82.1	82.3
Calanoida	69 405.1 (5886.8)	46 224.8 (5191.7)	39.2	41.3
Cyclopoida	70 280.5 (5830.4)	42 480.8 (4706.5)	39.7	37.9
Harpacticoida	5541.9 (987.5)	3533.5 (512.3)	3.1	3.2
Monstrilloida	1.9 (1.9)	6.9 (5.1)	<0.01	<0.01
**non-Copepod groups**	31 729.8 (2648.5)	19 813.5 (3001.5)	17.9	17.7
Amphipoda	77.0 (24.0)	39.3 (20.7)	0.04	0.04
Appendicularia	6179.3 (671.2)	5436.3 (939.7)	3.5	4.9
Chaetognatha	2177.4 (358.7)	2157.0 (254.6)	1.2	1.9
Cnidaria				
Leptothecata	143.5 (66.6)	54.9 (18.3)	0.1	0.05
Hydromedusae unidn.^a^		0.6 (0.6)		<0.01
Siphonophorae	1037.5 (148.9)	754.9 (136.8)	0.6	0.7
Diplostraca				
Ctenopoda	6925.9 (1534.9)	2112.4 (537.1)	3.9	1.9
Onychopoda	2786.5 (608.9)	149.0 (43.4)	1.6	0.1
Decapoda – *Lucifer* spp.	377.3 (229.5)	236.0 (81.3)	0.2	0.2
Doliolida	2621.9 (688.5)	1774.1 (929.2)	1.5	1.6
Euphausiacea	931.9 (177.2)	979.5 (280.3)	0.5	0.9
Isopoda	23.7 (16.4)	50.9 (14.3)	0.01	0.05
Mysidacea	13.1 (6.2)	56.5 (21.2)	0.01	0.1
Ostracoda	1041.0 (299.1)	911.4 (265.5)	0.6	0.8
Pteropoda	2511.8 (501.2)	2862.4 (1179.7)	1.4	2.6
Salpida	126.4 (97.6)	147.8 (95.7)	0.1	0.1
**Meroplankton**				
Cirripedia larvae	1116.5 (452.7)	80.9 (24.2)	0.6	0.1
Decapoda larvae	2038.3 (288.3)	1153.0 (216.5)	1.2	1.0
Echinodermata larvae	41.5 (19.8)	111.2 (58.2)	0.02	0.1
Fish egg	613.5 (318.2)	279.6 (77.6)	0.3	0.2
Fish larvae	180.0 (46.9)	311.3 (72.0)	0.1	0.3
Phoronida	15.8 (12.4)		0.01	
Polychaeta larvae	746.0 (170.5)	149.7 (55.3)	0.4	0.1
Stomatopoda – *Squilla* spp.	4.0 (4.0)	4.9 (4.6)	<0.01	<0.01

The standard error is provided in parentheses.

^a^Assumed as holoplankton.

**Table III:** Mean abundance (ind. m^−2^) and the percent of positive stations (frequency of occurrence, %FO) of the copepod and diplostracan taxa identified in the samples collected in 2017 and 2019.

**Taxa**	**Mean Abundance**		**%FO**		**Taxa**	**Mean Abundance**		**%FO**	
**Calanoida**	**2017**	**2019**	**2017**	**2019**		**2017**	**2019**	**2017**	**2019**
*Acartia (Acanthacartia) tonsa*		25.4 (22.2)		2.9	*Candacia bipinnata*	3.9 (3.9)	35.8 (17.5)	1.2	8.6
*Acartia (Acartia) danae*	357.4 (70.2)	793 (210.4)	43.2	50.0	*Candacia elongata*	1.9 (1.9)	119.7 (75.2)	1.2	11.4
*Acartia (Acartia) negligens*		181 (161)		8.6	*Candacia longimana*		47.5 (44.0)		2.9
*Acartia (Acartiura) clausi*	1552.9 (251.5)	2389.9 (538.2)	56.8	54.3	*Candacia simplex*	35.6 (12.3)	18.3 (8.9)	11.1	7.1
*Acartia (Acartiura) discaudata*		15.2 (13.8)		2.9	*Candacia* spp.	110.6 (28.6)	32.8 (11.0)	21.0	14.3
*Acartia (Acartiura) longiremis*		75.6 (47.2)		5.7	*Candacia varicans*		19.2 (15.3)		2.9
*Acartia* spp.	2576.1 (507.7)	2934.4 (669.9)	79.0	64.3	*Centropages abdominalis*		15.2 (14.6)		2.9
*Acrocalanus* spp.	207.4 (73.2)	52.6 (30.0)	18.5	7.1	*Centropages bradyi*	61.2 (37.7)	43.4 (24.1)	8.6	7.1
*Aetideopsis multiserrata*		4.6 (4.6)		1.4	*Centropages chierchiae*	451.9 (133.6)	252.6 (123.3)	30.9	24.3
*Aetideopsis rostrata*		12.8 (12.8)		1.4	*Centropages velificatus*	300.2 (158.9)	648.4 (217.0)	14.8	27.1
*Aetideopsis* spp.	1.9 (1.9)		1.2		*Centropages hamatus*		20.1 (15.1)		4.3
*Aetideus armatus*	5.3 (4.1)	97.5 (73.8)	2.5	8.6	*Centropages* spp.	2699.8 (441.1)	1321.6 (290.3)	79.0	60.0
*Aetideus bradyi*	1.3 (1.3)		1.2		*Centropages typicus*	569.4 (158.5)	951.7 (289.6)	39.5	51.4
*Aetideus giesbrechti*	4.0 (4.0)	4.6 (4.6)	1.2	1.4	*Centropages violaceus*	11.9 (6.8)	29.3 (29.3)	3.7	1.4
*Aetideus* spp.	1.3 (1.3)		1.2		*Chiridius gracilis*		13.7 (7.1)		5.7
*Anomalocera* spp.	3.9 (3.9)		1.2		*Chiridius poppei*	11.9 (11.9)		1.2	
*Augaptilus megalurus*		1.8 (1.8)		1.4	*Chiridius* spp.	15.8 (9.6)	4.6 (4.6)	3.7	1.4
*Bradycalanus* spp.		4.6 (4.6)		1.4	*Clausocalanus arcuicornis*	253.5 (64.5)	770.2 (230.5)	23.5	42.9
*Calanoida unidn.^b^*	177.8 (42.5)	237.7 (83.4)	28.4	32.9	*Clausocalanus furcatus*	3979.6 (578.1)	4109.9 (604.4)	85.2	92.9
*Calanoides natalis*	2547.7 (995.7)	888.3 (285.1)	34.6	44.3	*Clausocalanus paululus*	11.9 (11.9)		1.2	
*Calanus* spp.	802.5 (156.0)	2287.2 (558.9)	61.7	87.1	*Clausocalanus pergens*		1.8 (1.8)		1.4
*Calocalanus contractus*		33.8 (18.7)		5.7	*Clausocalanus spp.*	10554.2 (1145.6)	3720.3 (497.6)	91.4	80.0
*Calocalanus pavo*	1287.2 (175.1)	1434.8 (231.7)	65.4	75.7	*Ctenocalanus vanus*		2.4 (1.7)		2.9
*Calocalanus plumulosus*		18.3 (16.1)		2.9	*Diaixis gambiensis*	69.1 (26.2)	22.9 (11.9)	11.1	5.7
*Calocalanus* spp.	578.8 (102.0)	412.8 (85.7)	46.9	38.6	*Diaixis hibernica*		18.3 (18.3)		1.4
*Calocalanus styliremis*	3.9 (3.9)	94.3 (47.1)	1.2	10.0	*Diaixis pygmaea*	59.3 (17.7)	61.0 (22.3)	16.0	14.3
*Calocalanus tenuis*	76.4 (20.8)	90.1 (27.9)	19.8	17.1	*Diaixis* spp.	128.4 (34.5)	53.6 (22.5)	19.8	11.4
*Candacia ethiopica*		140.2 (131.8)		2.9	*Euaugaptilus* spp.		1.5 (1.5)		1.4
*Candacia armata*		24.1 (14.5)		5.7	*Eucalanus hyalinus*	11.9 (8.8)		2.5	
*Euchaeta acuta*	83.6 (46.6)	105.9 (42.5)	9.9	11.4	*Microcalanus pusillus*	1.3 (1.3)	124.0 (41.3)	1.2	18.6
*Euchaeta concinna*	3.9 (3.9)	11.4 (6.8)	1.2	4.3	*Microcalanus* spp.	13.8 (7.0)	132.1 (46.1)	4.9	21.4
*Paraeuchaeta hebes*		11.0 (11.0)		1.4	*Nannocalanus minor*	256.6 (45.0)	528.9 (150.9)	40.7	47.1
*Euchaeta marina*	290.4 (75.3)	252.1 (65.4)	21.0	28.6	*Neocalanus gracilis*	108.6 (31.0)	1148.3 (666.8)	17.3	32.9
*Euchaeta pubera*	5.9 (4.4)	55.5 (20.4)	2.5	14.3	*Paracalanus parvus^a^*	18300.1 (2638.7)	8739.3 (1413.5)	95.1	100.0
*Euchaeta spinosa*		21.6 (12.7)		5.7	*Paracartia grani grani*		5.0 (3.5)		2.9
*Euchaeta* spp.	851.4 (228.1)	449.0 (118.7)	37.0	34.3	*Paraeuchaeta spp.*	7.9 (7.9)		1.2	
*Gaetanus brevispinus*		1.8 (1.8)		1.4	*Parapontella brevicornis*		3.7 (3.7)		1.4
*Gaetanus kruppii*	7.9 (7.9)		1.2		*Pareucalanus* spp.	229.1 (160.5)	40.7 (18.3)	9.9	11.4
*Gaetanus minor*	3.9 (3.9)		1.2		*Pleuromamma gracilis*	52.0 (21.7)	367.8 (135)	9.9	37.1
*Gaetanus* spp.	19.8 (12.7)		3.7		*Pleuromamma piseki*		7.3 (7.3)		1.4
*Haloptilus acutifrons*	7.9 (5.6)		2.5		*Pleuromamma robusta*		91.3 (47.7)		15.7
*Haloptilus longicornis*	19.8 (10.3)	41.1 (19.2)	4.9	8.6	*Pleuromamma* spp*.*	176.5 (55.9)	199.9 (59.0)	22.2	22.9
*Haloptilus oxycephalus*		2.3 (2.3)		1.4	*Pleuromamma xiphias*	1.3 (1.3)	16.5 (14.7)	1.2	2.9
*Haloptilus* spp.	21.1 (8.7)	18.4 (12.0)	7.4	5.7	*Pontella atlantica*	9.9 (9.9)		1.2	
*Isias clavipes*	39.5 (28.3)	14.6 (11.5)	2.5	2.9	*Pontella* spp.				
*Labidocera acutifrons*	11.9 (6.8)		3.7		*Pontellina plumata*	11.9 (8.8)	5.7 (4.1)	2.5	2.9
*Labidocera nerii*	15.8 (9.6)		3.7		*Pseudocalanus elongatus*		37.6 (23.9)		7.1
*Labidocera* spp.	37.5 (13.8)	32.0 (27.7)	9.9	2.9	*Pseudocalanus minutus*		13.7 (13.7)		1.4
*Labidocera wollastoni*	3.9 (3.9)	4.6 (4.6)	1.2	1.4	*Pseudocalanus* spp.	11.9 (11.9)	64.9 (44.3)	1.2	4.3
*Lucicutia clausi*		1.5 (1.5)		1.4	*Pseudophaenna typica*	15.8 (15.8)		1.2	
*Lucicutia curta*		0.6 (0.6)		1.4	*Rhincalanus cornutus*	13.8 (7.0)		4.9	
*Lucicutia flavicornis*	247.6 (54.0)	275.2 (57.9)	33.3	40.0	*Rhincalanus nasutus*		50.6 (30.1)		7.1
*Lucicutia longiserrata*		6.9 (6.9)		1.4	*Scottocalanus persecans*		0.6 (0.6)		1.4
*Mecynocera clausi*	565.6 (139.6)	369.0 (97.8)	34.6	41.4	*Spinocalanus* spp.	1388.1 (277.4)	727.5 (119.1)	81.5	68.6
*Megacalanus princeps*		20.3 (9.9)		7.1	*Subeucalanus* spp.	3283.0 (1042.1)	2866.7 (476.1)	63.0	80.0
*Mesocalanus tenuicornis*	15.8 (11.1)	68.0 (40.2)	2.5	7.1	*Talacalanus greenii*		1.8 (1.8)		1.4
*Metridia lucens*		5.8 (4.7)		2.9	*Temora longicornis*	108.6 (50.4)	1382.6 (791.7)	7.4	24.3
*Metridia* spp.		6.7 (4.8)		4.3	*Temora* spp.	8807.2 (1669.4)	1176.6 (289.4)	84.0	64.3
*Metridia venusta*		22.6 (11.6)		7.1	*Temora stylifera*	3631.6 (678.5)	1744.8 (359.1)	81.5	72.9
*Microcalanus pygmaeus*	86.9 (27.7)	118.6 (33.9)	13.6	21.4	*Temora turbinata*	1108.1 (683.1)	213.7 (108.3)	11.1	17.1
*Undeuchaeta plumosa*		5.7 (3.4)		4.3	*Sapphirina* spp.	106.7 (67.5)	276.1 (140.3)	8.6	24.3
*Undeuchaeta* spp.		9.1 (9.1)		1.4	**Harpacticoida**				
*Xanthocalanus hirtipes*		9.1 (9.1)		1.4	*Aegisthus aculeatus*		2.9 (2.9)		1.4
*Xantocalanus* spp.		2.3 (2.3)		1.4	*Clytemnestra gracilis*	27.7 (11.9)	24.0 (11.7)	7.4	7.1
**Cyclopoida**					*Clytemnestra scutellata*		19.4 (14.9)		4.3
*Copilia mirabilis*	7.9 (5.6)	8.4 (5.3)	2.5	4.3	*Clytemnestra* spp.	59.3 (23.8)	10.3 (6.5)	9.9	4.3
*Copilia quadrata*	3.9 (3.9)	39.3 (23.4)	1.2	7.1	*Euterpina acutifrons*	4225.8 (990.9)	2116.6 (506.7)	69.1	60.0
*Copilia* spp.	13.8 (7.0)	13.7 (7.8)	4.9	4.3	*Harpacticoida unidn.^b^*	71.1 (37.2)		7.4	
*Corycaeidae*	3766.1 (594.2)	3115.4 (349.5)	88.9	95.7	*Macrosetella gracilis*	365.4 (88.2)	768.4 (205.7)	28.4	37.1
*Cyclopoida* unidn.^b^		17.1 (17.1)		1.4	*Microsetella norvegica*	190.3 (65.9)	111.8 (41.1)	24.7	17.1
*Lubbockia aculeata*	3.9 (3.9)	13.7 (11.6)	1.2	2.9	*Microsetella rosea*	392.9 (75.2)	360.2 (56.9)	45.7	60.0
*Lubbockia squillimana*	99.4 (25.9)	71.6 (17.7)	19.8	24.3	*Microsetella* spp.	197.5 (52.8)	91.4 (36.6)	30.9	18.6
*Oithona nana*	6598.0 (1521.3)	5547.7 (1181.6)	69.1	78.6	*Miracia efferata*	11.9 (7.3)	28.5 (20.8)	3.7	7.1
*Oithona plumifera*	2407.2 (302.2)	2305.8 (298.5)	84.0	88.6	**Monstrilloida**				
*Oithona* spp.	15935.5 (1602.0)	6349.8 (895.7)	97.5	91.4	*Monstrilla* spp.	1.9 (1.9)	6.9 (5.1)	1.2	2.9
*Oncaea curta*	14964.1 (2881.6)	5612.8 (1833.8)	69.1	67.1	**Diplostraca**				
*Oncaea venusta*	5436.7 (600.8)	12509.0 (2243.6)	91.4	98.6	*Penilia avirostris*	7093.2 (1549.9)	2112.4 (537.1)	67.9	55.7
*Oncaeidae*	20937.1 (2448.6)	6600.5 (1003.3)	100.0	87.1	*Podon* spp.	2771.9 (614.7)	149.0 (43.4)	62.9	22.9

The standard error is provided in parentheses.

^a^ *P. parvus* species complex;

^b^unidn.: unidentified taxon

**Fig. 5:**
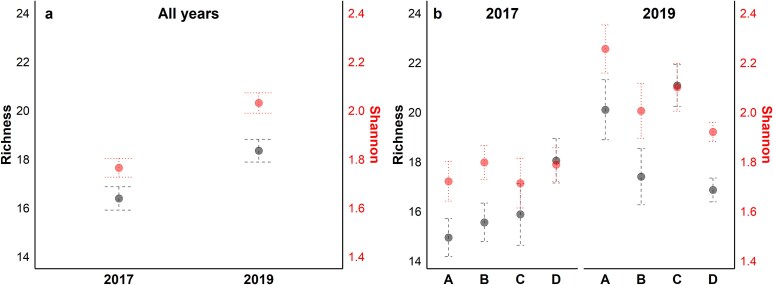
Mean taxonomic richness (left y-axis) and Shannon-Weiner index (right y-axis) calculated at the genus level for (**a**) 2017 and 2019 and for (**b**) the upwelling Zones (A, B, C, D) for each year. Vertical bars indicate standard errors. The number of stations for each zone is the same as in Fig. 4c.

Calanoids and cyclopoids primarily composed the copepod assemblage (Table II, Fig. S3). Important and broadly distributed copepod taxa in the region were *Oithona* spp. Oncaeidae, Corycaeidae, *Clausocalanus furcatus*, *Acartia danae*, Paracalanus parvus^*^ and *Temora stylifera* (Fig. S4). Interestingly, different distribution patterns were observed among congeners (e.g. genera of *Acartia, Centropages*, *Oithona*, *Temora*), while some taxa (e.g. *Oithona curta*, *Acartia clausi*, *Centropages typicus*, *Oithona nana*) were mostly recorded north of Cape Blanc (Fig. S5a), some tropical taxa (e.g. *Centropages velificatus*, *Macrosetella gracilis*, *Temora turbinata*, *Acrocalanus* spp.) were found southern of Cape Blanc (Fig. S5b). Both taxonomic richness and Shannon index were found higher in 2019 with distinct latitudinal variations between years (Fig. 5). PERMANOVA analysis showed significant differences in the assemblage structure across Zones and strata for each year (Table I). Zone D differed markedly from the others in 2017, while differences across all Zones were observed in 2019 (Table S2). The inshore assemblage (Str1) differed significantly from the other strata in 2017, while in 2019, differences were detected against stations further offshore (Str3) (Table S2).

**Fig. 6:**
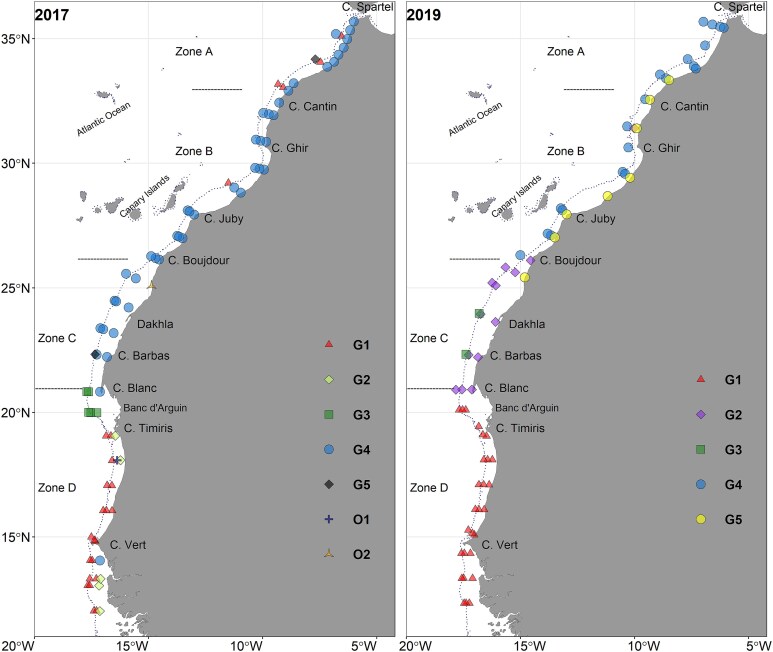
Distribution maps of the cluster groups defined by the hierarchical clustering in 2017 and 2019 (O1, O2: outliers stations shown in Fig. S6).

**Fig. 7:**
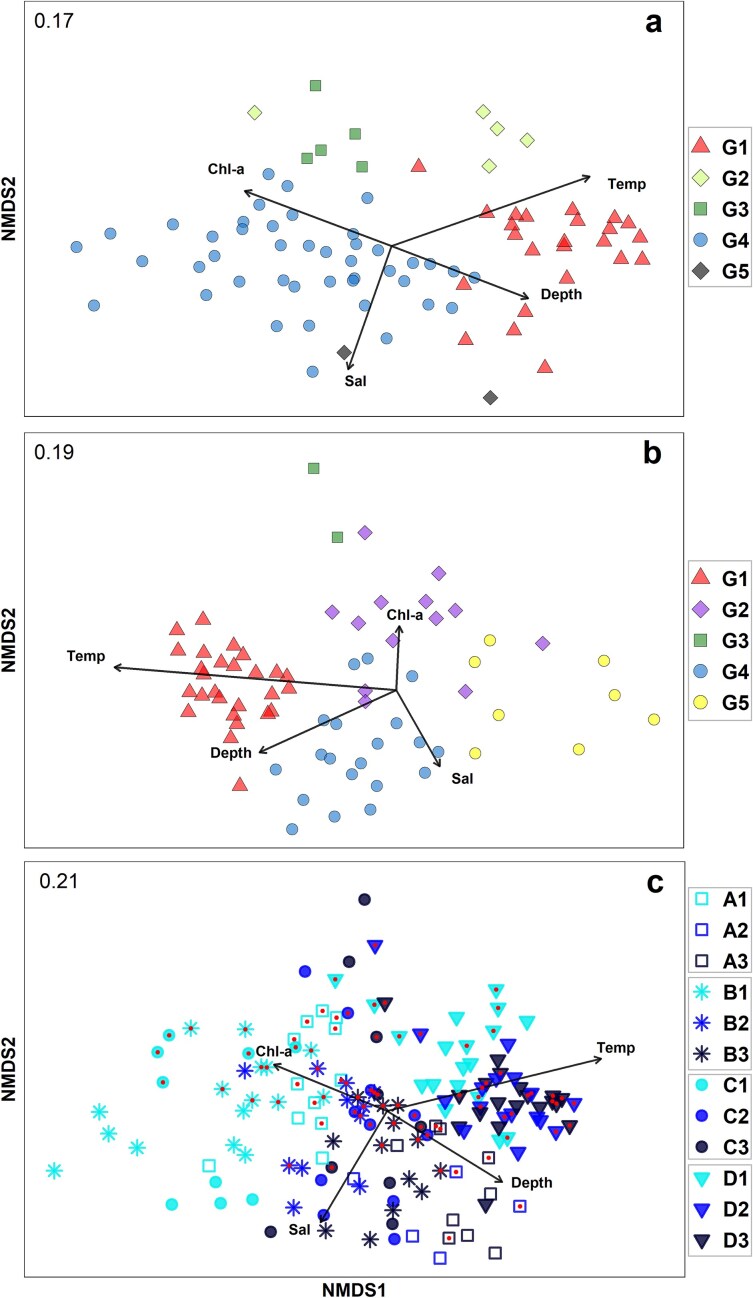
Ordination plots of Non-Metric Multi-Dimensional Scaling (nMDS) for (**a**) 2017, (**b**) 2019, and (**c**) the combined data of 2017 and 2019 based on Bray–Curtis distance and square-root transformed abundance data of copepods and diplostracans (stress value is given on the top left). Environmental vectors (Temp: temperature, Sal: salinity, Chl-a: Chlorophyll-a, Depth: sampling depth) fitted to nMDS and having a significant correlation (*P* < 0.05) as identified with the envfit function are shown. Information of the cluster groups (G1–G5) has been superimposed in (a) and (b). Information of the combination of the upwelling Zones (A, B, C, D) and the bathymetric strata (1, 2, 3) have been superimposed in (c). Outlier stations have been omitted from the ordination. Stations sampled in 2017 have been distinguished by dots inside the symbols.

**Fig. 8:**
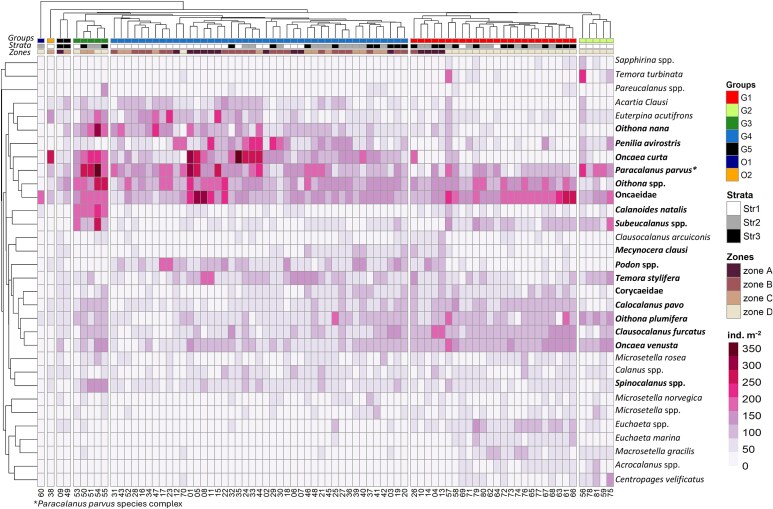
Heatmap of the square-root transformed abundances (ind. m^−2^) for taxa accounting for > 3% of the copepod and diplostracan total abundance in 2017. The colors indicate abundance values (ind m^−2^). The colored bars indicate groups of stations (Groups) identified by cluster analysis (Fig. S6), the bathymetric strata (1, 2, and 3), and the distinct upwelling latitudinal Zones (A, B, C, D). Copepod and diplostracan are clustered (group average) based on the Bray–Curtis distance matrix of their relative abundance. Bold font refers to taxa contributing to 70% similarity described in the SIMPER Table S5. Numbers along the bottom of the heatmap correspond to the station number as shown in Figs S1 and S6.

**Fig. 9:**
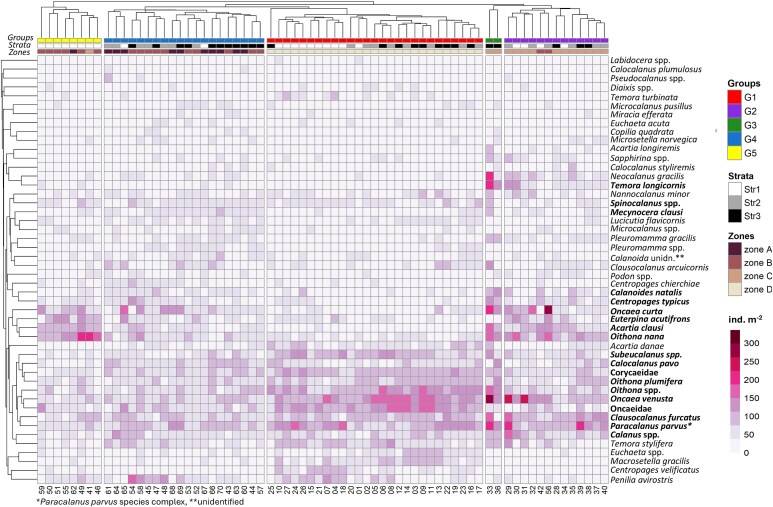
Heatmap of the square-root transformed abundances (ind. m^−2^) for taxa accounting for > 3% of the copepod and diplostracan total abundance in 2019. The colors indicate abundance values (ind m^−2^). The colored bars indicate groups of stations (Groups) identified by cluster analysis (Fig. S6), the bathymetric strata (1,2, and 3), and the distinct upwelling latitudinal Zones (A, B, C, D). Copepod and diplostracan are clustered (group average) based on the Bray–Curtis distance matrix of their relative abundance. Bold font refers to taxa contributing to 70% similarity described in the SIMPER Table S6. Numbers along the bottom of the heatmap correspond to the station number as shown in Figs S1 and S6.

Hierarchical clustering further highlighted the spatial differentiation in the assemblage structure along the northwest African coast in both years (Figs 6 and S6). The station grouping, also evidenced in the nMDS ordination (Fig. 7), reflected latitudinal changes, which were more pronounced in 2019. In spring/summer 2017, five main clusters were found at 51% dissimilarity (Fig. S6). Stations located South of Cape Blanc (Zone D) were clustered in three distinct groups (G1-G3), with G1 representing their majority (n = 19), plus five stations in Str3 north of Canary Islands (Fig. 6). Cluster G2 comprised five coastal stations, while five others between Cape Blanc and Cape Timiris (19°N), influenced by frontal waters, formed G3 (Fig. 6). Stations north of Cape Blanc (n = 43) formed G4 (n = 43), except two offshore stations in Zones A and C (G5 Group). Figure 8 presents the associations of major taxa (>3% relative abundance) and their contribution to the station clusters. SIMPER analysis results are listed in Table S5. Oncaeidae, *Oithona* spp., Oncaea venusta and P. parvus^*^ were important across all clusters (Fig. 8, Table S5). However, *C. furcatus*, Corycaeidae and *Oithona plumifera* were more influential in clusters South of Cape Blanc, while sites at the frontal area (G3) exhibited overall high abundances of *Calanoides natalis*. In 2019, cluster analysis revealed five major station groups at 56% dissimilarity (Fig. S6). All stations sampled South of Cape Blanc (n = 27) were grouped in one cluster, G1 (Fig. 6). Samples collected within the permanent strong upwelling Zone C were grouped as G2 cluster (n = 13), except two offshore sites of higher abundances and diversity that formed G3 (Figs S6 and S7). North of Cape Bojador (Zones A, B) was separated into two clusters: one offshore with stations mainly located at Str2 and Str3 (G4, n = 20), and one coastal at Str1 (G5; n = 8) characterized by a neritic assemblage of *O. nana, Euterpina acutifrons* and *A. clausi* (Fig. 9, Table S6). Taxa such as Oncaeidae, *O. nana*, *O. venusta*, *Oncaea curta* and *A. clausi* made the major differences among the clusters identified north and south of Cape Blanc (Fig. 9, Table S6).

The output of multiple regressions done through the *envfit* function is provided in Table IV, and the averaged environmental parameters per group in Table V. All parameters significantly correlated with the nMDS ordination scores in both years apart, except for the oxygen (Fig. 7a and b). In 2017, temperature differentiated stations north and south of Cape Blanc (G4 vs. G1, G2), while salinity distinguished the clusters associated with the frontal Zone and riverine outflow, G2 and G3, respectively. Depth and Chl-a mainly appeared related to the differentiation of G1 from G4 and G3. In 2019, temperature also explained strongly the separation of stations south of Cape Blanc (G1) from the rest. Notably, Chl-a was related to the differentiation of stations in Zone C (G2, G3), while salinity mainly explained the differentiation of the clusters north of Cape Bojador (G4 and G5). Sampling depth mostly explained the differentiation of coastal stations north of Cape Bojador (G5). The nMDS ordination on all samples revealed the similarity of stations South of Cap Blanc in both years (Zone D) strongly associated with the increase in temperature (Fig. 7c). Depth and Chl-a mostly explained the differentiation of inshore stations (Str1) from other strata across all Zones in both years. Salinity was associated with the differentiation of stations in deeper waters (Str2, 3), particularly in Zones B and C.

**Table IV:** Fits of selected environmental vectors to the nMDS ordination of the copepod and diplostracan assemblage structure.

**Vectors**	**nMDS1**	**nMDS2**	***r*** ^***2***^	**Pr (> r)**	**p adj.**
**2017**					
Temp	0.86484	0.50205	0.7238	**0.001**	0.005^**^
Sal	−0.2092	−0.9779	0.6006	**0.001**	0.005^**^
Chl-a	−0.8487	0.5289	0.4112	**0.001**	0.005^**^
Oxy	−0.0621	−0.9981	0.0781	**0.042**	**0.21**
Depth	0.84493	−0.5349	0.3591	**0.001**	0.005^**^
**2019**					
Temp	−0.9928	0.11946	0.7193	**0.001**	0.005^**^
Sal	0.7407	−0.6718	0.2586	**0.001**	0.005^**^
Chl-a	0.6177	0.78641	0.1322	**0.009**	0.005^**^
Oxy	−0.0437	−0.999	0.1052	**0.025**	**0.125**
Depth	−0.7297	−0.6837	0.1663	**0.002**	0.005^**^
**All years**					
Temp	0.67317	−0.73949	0.2939	**0.001**	0.005^**^
Sal	0.99028	0.13907	0.6722	**0.001**	0.005^**^
Chl-a	−0.51312	−0.85832	0.3173	**0.001**	0.005^**^
Oxy	−0.81337	0.58175	0.2255	**0.099**	**0.52**
Depth	0.35057	−0.93653	0.0303	**0.001**	0.005^**^

Environmental vectors: mean Temperature (Temp), Salinity (Sal), Chl-a, Oxygen (Oxy) in the 0–30 m layer and station sampling depth (Depth). r^2^: Correlation coefficient and Pr(>r): P-value. P-values were adjusted (p adj.) using the Bonferroni method to account for multiple testing, with significance levels indicated by p < 0.01(^**^)

**Table V:** Mean temperature (Temp), salinity (Sal), Chlorophyll-a (Chl-a), Oxygen in the 0–30 m sampling layer (Oxy), and the sampling depth of the station groups defined by the cluster analysis in 2017 and 2019 (O1, O2: outliers stations shown in Fig. S6).

**2017**	**G1**	**G2**	**G3**	**G4**	**G5**	**O1**	**O2**
Temp	25.9 (0.7)	27.0 (0.8)	20.2 (0.2)	19.0 (0.3)	20.6 (0.8)	26.7	16.0
Sal	36.1 (0.02)	35.9 (0.1)	35.8 (0.02)	36.3 (0.04)	36.5 (0.1)	36.0	36.2
Chl-a	0.3 (0.04)	0.7 (0.1)	2.6 (0.5)	1.1 (0.2)	0.2 (0.1)	0.5	1.3
Oxy	4.7 (0.1)	4.6 (0.1)	4.8 (0.2)	4.9 (0.1)	5.2 (0.1)	4.5	3.9
Depth	142.3 (12.6)	26.0 (1.9)	122.0 (33.7)	84.2 (10.7)	200.0	100.0	40.0
N	24	5	5	43	2	1	1
**2019**	**G1**	**G2**	**G3**	**G4**	**G5**		
Temp	26.5 (0.4)	19.4 (0.6)	19.7 (0.5)	18.7 (0.3)	16.2 (0.4)		
Sal	35.8 (0.1)	36.3 (0.1)	36.3 (0.1)	36.5 (0.03)	36.2 (0.1)		
Chl-a	0.7 (0.1)	1.5 (0.4)	2.4 (1.3)	1.0 (0.3)	0.8 (0.1)		
Oxy	4.2 (0.1)	4.4 (0.3)	4.9 (0.2)	5.2 (0.02)	4.0 (0.3)		
Depth	105.7 (14.2)	99.0 (18.3)	200.0	145.9 (14.4)	26.3 (0.8)		
N	27	13	2	20	8		

The standard error is provided in parentheses. N: number of stations.

## DISCUSSION

Our work revealed significant latitudinal and seasonal/interannual variability in mesozooplankton stock and assemblage structure along the Northwest African coast, closely linked to the dominant oceanographic conditions of the CCLME. In both sampling periods (spring/summer 2017 and autumn/winter 2019), we observed a pronounced shift in the oceanographic regime at the Cape Blanc boundary (21°N) off Mauritania, linked to the convergence of NACW and SACW at the Cape Verde frontal Zone (Zenk *et al.*, 1991; Pérez-Rodríguez *et al.*, 2001). This appeared closely tied to the upwelling dynamics prevailing in each subregion on a seasonal basis, as documented by previous studies (Arístegui *et al.*, 2009; Cropper *et al.*, 2014).

Permanent upwelling activity, albeit with variations in intensity depending on latitude and season, characterizes Zones B and C north of Cape Blanc (e.g. Arístegui *et al.*, 2009; Cropper *et al.*, 2014). In both years, we observed lower temperatures and high Chl-a inshore, indicating the presence of cooler, productive, upwelled waters in areas with bathymetry 0–50 m (Benazzouz *et al.*, 2014b). In spring/summer 2017, Zones B and C showed similar hydrological conditions, and offshore waters (Str2, 3) exhibited higher temperatures and stronger stratification, than in late autumn/winter 2019. Mesozooplankton stock was found to be overall higher during this sampling period, likely linked to the seasonal upwelling dynamics and partly also to the diplostracan distribution known for their population explosions during warmer periods in temperate ecosystems (e.g. Isari *et al.*, 2007; Atienza *et al.*, 2016). Summer marks the time of year when seasonal upwelling intensity north of Cape Bojador (26^o^N) is expected to intensify (e.g. Arístegui *et al.*, 2009; Cropper *et al.*, 2014; Benazzouz *et al.*, 2014a). Wind stress and upwelling activity typically strengthen around the primary upwelling centres of Cape Juby (27^o^N) and Cape Ghir (31^o^N) (Marcello *et al.*, 2011), with a filament development around these capes to peak during summer (Marcello *et al.*, 2011), and therefore enhancing offshore organic export (Pelegrí *et al.*, 2005). Interestingly, in late autumn/winter 2019, a strong band of upwelled cooler waters emerged along the Moroccan coastline between Cape Bojador and Cape Cantin (31^o^N) (Zone B) marked by elevated Chl-a and oxygen concentrations. Nevertheless, this unusual upwelling event for the season was not reflected on the mesozooplankton stock during our surveys, likely due to either time lag in mesozooplankton response to lower trophic levels, or more complex food web processes, including prey–predator interactions.

The region from north of Cape Blanc to Cape Bojador (Zone C), characterized by a broad and shallow continental shelf, experiences strong year-round upwelling *(*Arístegui *et al.*, 2009; Fréon *et al.*, 2009; Cropper *et al.*, 2014; Benazzouz *et al.*, 2014a) and serves as an optimal spawning, and nursery ground for small pelagic fish species (Ettahiri *et al.*, 2003; Brochier *et al.*, 2018). During the current work, this area south of Morocco exhibited relatively higher zooplankton stocks than other Zones north of Cape Blanc (A and B). In summer, no strong differences in assemblage structure were observed north of Cap Blanc in line with the similarity of hydrological conditions among Zones. Notably, the stronger hydrographic variability encountered among Zones in 2019, was clearly reflected on the station clustering. Zone C was characterized by a distinct and more diverse assemblage, likely influenced by higher Chl-a concentrations, while stations north of Cape Bojador (Zones A and B) were separated based on station depth.

In contrast, the southern CCLME (Zone D) experiences large seasonal variability in upwelling activity (Arístegui *et al.*, 2009; Cropper *et al.*, 2014) primarily occurring in winter due to the northern migration of the Intertropical Convergence Zone (Cropper *et al.*, 2014; Sylla *et al.*, 2019). Acoustic surveys across the CCLME have reported higher plankton and fish abundances in this sub-region (Diogoul *et al.*, 2021), while the high levels of productivity south of Cape Blanc are attributed to the combined influence of the nutrient-rich SACW, high rates of deposition of Sahara Dust and freshwater runoff (Arístegui *et al.*, 2009). Our sampling in both years, taking place during the upwelling relaxation in Zone D, revealed an overall southward increase in mesozooplankton stock. The warmer and well-stratified waters south of Cape Blanc exhibited overall higher mesozooplankton stock and distinct taxonomic composition compared to the north. In 2017, higher values of abundance and dry weight were observed, and also stronger spatial variability in assemblage structure, largely linked to salinity gradients and high Chl-a levels at the convergence Zone (off Banc d’Arguin) and at the southern tip of the CCLME. Seasonal/interannual variations of mesozooplankton during the monsoon season off Mauritania are associated with shifts of the northern boundary of SACW (Sirota *et al.*, 2004), while intensive development around Cape Blanc particularly in winter (Kuipers *et al.*, 1993) is linked to the Senegal-Mauritanian front and the coastal upwelling. Such processes highlight the role of local productivity hotspots within the Mauritanian-Senegalese system, essential for the recruitment of small pelagic fish (Guénette *et al.*, 2014; Brochier *et al.*, 2018; Tiedemann *et al.*, 2018). Similarly, low salinity waters over the extended shelf south of Cape Vert in 2017, likely connected with intensification of the onshore monsoonal winds, precipitation and freshwater runoff (Gómez-Letona *et al.*, 2017), shaped a distinct neritic assemblage as previously observed by Lidvanov *et al.* (2022).

Cape Blanc is recognized as a boundary for tropical copepod species extending north and for temperate/subtropical extending south (Berraho *et al.*, 2015), as well as a general faunistic limit for planktonic communities (Hamann *et al.*, 1981; Weikert, 1982). In both years of the study, increased temperatures south of Cape Blanc emerged as the main driver of the shift in assemblage structure. Copepod taxa associated with the northward advection of SACW (Sirota *et al.*, 2004; Glushko and Lidvanov, 2012) were prominent in Zone D, but were nearly absent north of Cape Blanc, aligning with previous observations along the Moroccan coast (Lidvanov *et al.*, 2013). Observed differences during our work in the latitudinal distribution patterns among taxa, including congeners, reflect taxon-specific environmental preferences (e.g. thermal window) and ecophysiological traits (Hays *et al.*, 2005; Richardson, 2008). Commonly found in upwelling waters of western Africa (Verheye *et al.*, 2005; Wiafe *et al.*, 2008; Bode *et al.*, 2014) and associated with the SACW, the species *Calanoides natalis* (formerly identified as *C. carinatus*; Bradford-Grieve *et al.*, 2017) showed affinity for frontal waters near Cape Blanc but also a broader northern distribution in the CCLME, likely facilitated by the Canary Undercurrent (Postel *et al.,* 1995) in agreement with other observations in the region (e.g. Kuipers *et al.*, 1993; Hernández-León *et al.*, 2007; Salah *et al.*, 2012; Lidvanov *et al.*, 2018).

Overall, our results highlight the influence of seasonal upwelling dynamics along CCLME on the zooplankton, with increased standing stock and lower taxonomic richness and diversity in spring/summer 2017. The latitudinal upwelling Zones represent approximate boundaries that may vary over the years due to the meridional shift of the upwelling winds (Gómez-Letona *et al.*, 2017). Thus, there is a sub-regional variability in the mesozooplankton assemblage structure that is dynamically influenced by the upwelling and associated mesoscale activity. Variations in the position of the convergence Zone of the water masses as well as other mesoscale processes shape the spatial variability of environmental parameters and subsequently influence the ranges of distribution of the different species among seasons and years. This was reflected both on the migration of Chl-a maxima around Cape Blanc in the two sampling periods and the grouping of stations within different Zones based on their assemblage structure. An important seasonal signal in total zooplankton biomass characterized by higher values in upwelling periods and often changes in communities’ structures has been found for other EBUS as Benguela (Verheye *et al.*, 2016), Humboldt (e.g. Aronés *et al.*, 2019; Medellín-Mora *et al.*, 2020) and California (Mackas *et al.*, 2001, 2006). Interannual variation through multi-year time series has shown significant anomalies in biomass and community structure related to El Niño events (Mackas *et al.*, 2006; Lavaniegos and Ohman, 2007), strong shifts in size spectra with smaller copepod to dominate the assemblage (Verheye *et al.*, 2016), or, in other cases, no clear trends (Medellín-Mora *et al.*, 2020).

Unfortunately, due to the absence of monitoring programs in the CCLME (Berraho *et al.*, 2015) and the lack of regional studies, our understanding of long-term zooplankton dynamics in the region is rather poor. Our work is the first to explore the regional variability in mesozooplankton assemblage structure across all CCLME, while studies dating back to expeditions since 1994 through African-Russian partnerships, focused either on the Moroccan coastal waters (i.e. Somoue *et al.*, 2005; Lidvanov *et al.*, 2018), or on the south of Cape Blanc (Sirota *et al.*, 2004; Glushko and Lidvanov, 2012; Lidvanov *et al.*, 2022). Differences and/or uncertainties in the methods for sample collection and processing, the taxonomic resolution, but also the language used in several of these publications (i.e. Russian) pose serious challenges in meaningful comparisons within the CCLME. Our study is based on 180 μm-WPII net collections down to 200 m depth reflecting estimations of food availability for fish and their offspring within the CCLME epipelagic Zone. The mesozooplankton stock and assemblage structure at Str3 (station depth ≥ 500 m) within the study area may have been influenced, to some extent, by the light conditions during sampling. Stratified sampling in oceanic waters near the Canary Islands has shown evidence of DVM, with the > 1 mm fraction of mesozooplankton biomass (measured as protein content) migrating upwards to around 500 m during the day (Hernández-León *et al.*, 2001, 2007).

Recent studies from the broader CCLME region further demonstrate that the biomass migrating into the epipelagic layer at night is strongly linked to hydrological conditions and station-specific primary production (Hernández-León *et al.*, 2019, 2024). This suggests that the impact of DVM in Str3 may have varied latitudinally. However, the unbalanced sampling effort between day and night limited our ability to assess this effect. Notably, typical diel migrators such as euphausiids, had a small contribution in our WPII samples and were not included in the assemblage comparison across CCLME. Therefore, the impact of DVM only on the copepod-diplostracan assemblage could likely be considered minor, aligning with similar sampling challenges documented in other EBUS as discussed by Huggett *et al.* (2009). Assuming carbon content values to be 40% of the dry mass (Omori and Ikeda, 1984; Escribano *et al.*, 2007), mean values of the total mesozooplankton biomass across the CCLME in the current study ranged from 0.72 to 2.44 g C m^−2^ in 2017, and between 0.76 and 1.56 g C m^−2^ in 2019. These values are not only in concordance with the estimations reported for the area around the Canary Islands during the coastal upwelling season as well as for the open ocean (Hernández-León *et al.*, 2007; Couret *et al.*, 2023a), but also comparable to what has been reported for other EBUS as already summarized by Huggett *et al.* (2009) and Medellín-Mora *et al.* (2020).

Significant interannual trends in environmental variables, such as sea surface temperature, wind intensity, stratification and Chl-a, have been reported across CCLME, often showing varying responses at subregional scales (e.g. Arístegui *et al.*, 2009; Gómez-Letona *et al.*, 2017; Sylla *et al.*, 2019; Diogoul *et al.*, 2021; Vázquez *et al.*, 2023). While sea surface warming has impacted fish populations (Sarre *et al.*, 2024), studies on lower trophic levels, such as zooplankton, remain limited. Recent work by Couret *et al.* (2023b) documented a significant decline in mesozooplankton biomass around the Canary Islands, while Diogoul *et al.* (2021), found stable zooplankton abundance north and south of Cape Blanc using acoustic surveys. Nonetheless, we are still lacking knowledge about zooplankton assemblage structure, taxon-specific distribution, or size spectra in the region. To address these gaps, long-term and regional-scale monitoring efforts in CCLME must be enhanced, and inter-calibration efforts in taxonomic identification must be prioritized through strengthening intra-regional collaboration. In-situ broad-scale and spatially comprehensive data collection on lower trophic levels will help to better predict the responses of CCLME zooplankton communities to climate change.

## CONCLUSIONS

The present study indicates that upwelling dynamics and associated mesoscale processes as well as hydrological parameters variability influence the mesozooplankton standing stock and assemblage structure across the CCLME. Our results highlighted the presence of cooler productive upwelled waters along the shore north of Cape Blanc in both spring/summer 2017 and autumn/winter 2019, shaping the spatiotemporal mesozooplankton distribution patterns. Upwelling relaxation south of Cape Blanc resulted in warm and well stratified waters in both years characterized by high mesozooplankton stock and distinct species assemblages. Seasonality played an important role in modifying assemblage composition, with notable shifts in species dominance between sampling periods. Notably, Cape Blanc biogeographically served as a key boundary in both years; however, the mesozooplankton assemblages did not align strictly with the upwelling latitudinal zones but were more likely influenced by the complex hydrodynamics of the region.

## Supplementary Material

Supplementary_data_1_fbae079

Supplementary_data_2_fbae079

## Data Availability

The data were collected within the framework of the EAF-Nansen Programme and can be requested through the FAO webpage (https://www.fao.org/in-action/eaf-nansen/data-access-requests/en/). A metadata catalogue is available online to search for data used in this study and beyond (https://figisapps.fao.org/fishery/eafnansen/en/eafnansen/survey/search).
