# Supplementary material for: Spatio-temporal variability of mesozooplankton distribution along the Canary Current Large Marine Ecosystem: a regional perspective
Source: J Plankton Res. 2025 Jan 30;47(1):fbae079. doi: 10.1093/plankt/fbae079 (PMC11781819; doi:10.1093/plankt/fbae079)
Supplement: Supplementary_data_1_fbae079 [file supplementary_data_1_fbae079.docx]

# **SUPPLEMENTARY DATA 1**

## Table S1

| *Table S1. Sampling information of plankton and CTD stations for the surveys in 2017 and 2019.* | | | | | | | |
| --- | --- | --- | --- | --- | --- | --- | --- |
| **Year** | **Survey No** | **Latitudinal range** | **Sampling dates (DD/MM)** | **CTD stations** | **Plankton stations** | **Sampling time** | |
|  |  |  |  |  |  | **Day** | **Night** |
| **2017** | 2017401 | 36 - 21°N | 7/05 - 27/06 | 124 | 52 | 33 | 19 |
|  | 2017403 | 21 - 16°N | 28/06 - 9/07 | 35 | 15 | 6 | 9 |
|  | 2017404 | 16 - 12°N | 9/07 - 18/07 | 41 | 15 | 5 | 10 |
| **2019** | 2019411 | 16 - 12°N | 26/09 - 7/10 | 25 | 12 | 5 | 7 |
|  | 2019412 | 21 - 16°N | 9/10 - 20/10 | 24 | 15 | 12 | 3 |
|  | 2019413 | 33 – 21°N | 30/10 - 18/11 | 62 | 36 | 21 | 15 |
|  | 2019414 | 36 – 33°N | 23/11 - 28/11 | 21 | 13 | 7 | 6 |

## Table S2

| ***Table S2.*** *Pairwise comparisons of main effects (Zones: A, B, C, D; Strata:1,2, 3) following PERMANOVA analysis on (A) hydrological parameters at 10 m depth and (B) copepod and diplostraca assemblages in 2017 and 2019. P-values were adjusted (p adj.) using the Bonferroni method to account for multiple comparisons, with significance levels indicated by* ***p < 0.01(**)*** *and* ***p < 0.05(*)*** | | | | | | | |
| --- | --- | --- | --- | --- | --- | --- | --- |
| **Year** | **Pairwise comparisons** | **Df** | **Sum Of Sqs** | **R²** | **F** | **Pr(>F)** | **p adj.** |
| **A: Hydrology** | |  |  |  |  |  |  |
| **Zone/Stratum** | |  |  |  |  |  |  |
| **2017** | A_vs_B | 1 | 12.777 | 0.116 | 10.944 | 0.001 | **0.006**** |
|  | A_vs_C | 1 | 13.638 | 0.077 | 6.577 | 0.002 | **0.012*** |
|  | A_vs_D | 1 | 189.572 | 0.467 | 101.726 | 0.001 | **0.006**** |
|  | B_vs_C | 1 | 2.731 | 0.011 | 0.897 | 0.421 | 1 |
|  | B_vs_D | 1 | 204.191 | 0.408 | 80.735 | 0.001 | **0.006**** |
|  | C_vs_D | 1 | 158.018 | 0.303 | 49.215 | 0.001 | **0.006**** |
|  | 1_vs_3 | 1 | 26.268 | 0.041 | 6.121 | 0.003 | **0.009**** |
|  | 1_vs_2 | 1 | 30.230 | 0.057 | 7.427 | 0.001 | **0.003**** |
|  | 3_vs_2 | 1 | 3.315 | 0.008 | 1.071 | 0.317 | 0.951 |
|  |  |  |  |  |  |  |  |
| **2019** | A_vs_B | 1 | 9.432 | 0.091 | 6.178 | 0.001 | **0.006**** |
|  | A_vs_C | 1 | 33.460 | 0.215 | 10.404 | 0.001 | **0.006**** |
|  | A_vs_D | 1 | 129.089 | 0.656 | 129.733 | 0.001 | **0.006**** |
|  | B_vs_C | 1 | 15.591 | 0.069 | 4.476 | 0.008 | **0.048*** |
|  | B_vs_D | 1 | 162.130 | 0.512 | 94.465 | 0.001 | **0.006**** |
|  | C_vs_D | 1 | 85.077 | 0.319 | 30.847 | 0.001 | **0.006**** |
|  | 3_vs_2 | 1 | 5.090 | 0.020 | 1.898 | 0.126 | 0.378 |
|  | 3_vs_1 | 1 | 30.266 | 0.079 | 7.204 | 0.001 | **0.003**** |
|  | 2_vs_1 | 1 | 21.509 | 0.054 | 4.740 | 0.004 | **0.012*** |
|  |  |  |  |  |  |  |  |
| **B: Zooplankton** | |  |  |  |  |  |  |
| **2017** | A_vs_B | 1 | 0.216 | 0.054 | 1.994 | 0.060 | 0.36 |
|  | A_vs_C | 1 | 0.229 | 0.063 | 1.882 | 0.067 | 0.402 |
|  | A_vs_D | 1 | 0.865 | 0.143 | 7.005 | 0.001 | **0.006**** |
|  | B_vs_C | 1 | 0.126 | 0.033 | 1.190 | 0.269 | 1 |
|  | B_vs_D | 1 | 1.647 | 0.231 | 14.731 | 0.001 | **0.006**** |
|  | C_vs_D | 1 | 1.166 | 0.186 | 9.617 | 0.001 | **0.006**** |
|  | 1_vs_3 | 1 | 1.415 | 0.178 | 11.928 | 0.001 | **0.003**** |
|  | 1_vs_2 | 1 | 0.845 | 0.108 | 6.515 | 0.001 | **0.003**** |
|  | 3_vs_2 | 1 | 0.139 | 0.024 | 1.155 | 0.280 | 0.84 |
|  |  |  |  |  |  |  |  |
| **2019** | D_vs_C | 1 | 1.648 | 0.299 | 16.671 | 0.001 | **0.006**** |
|  | D_vs_B | 1 | 1.905 | 0.289 | 17.511 | 0.001 | **0.006**** |
|  | D_vs_A | 1 | 1.074 | 0.248 | 11.884 | 0.001 | **0.006**** |
|  | C_vs_B | 1 | 0.803 | 0.157 | 5.592 | 0.001 | **0.006**** |
|  | C_vs_A | 1 | 0.757 | 0.208 | 6.035 | 0.001 | **0.006**** |
|  | B_vs_A | 1 | 0.497 | 0.118 | 3.624 | 0.002 | **0.012*** |
|  | 1_vs_2 | 1 | 0.402 | 0.056 | 2.535 | 0.019 | 0.057 |
|  | 1_vs_3 | 1 | 0.805 | 0.099 | 5.190 | 0.001 | **0.003**** |
|  | 2_vs_3 | 1 | 0.182 | 0.028 | 1.256 | 0.214 | 0.642 |
|  | | | | | | | |

## Table S3

| ***Table S3.*** *Pairwise comparisons of the interaction between levels for Zones (A, B, C, D) and Strata (1,2, 3) for hydrological parameters at 10 m depth in 2017 and 2019. The comparisons are based on significant interactions identified from the PERMANOVA analysis. Bonferroni Adjusted p-values (p adj.) are shown, with significance levels indicated by* ***P < 0.05*** *(*).* | | | | | | | | |
| --- | --- | --- | --- | --- | --- | --- | --- | --- |
| **Year** | **Level** | **Pairwise comparisons** | **Df** | **Sum Of Sqs** | **R²** | **F** | **Pr(>F)** | **p adj.** |
| **2017** | A | 3_vs_2 | 1 | 2.319 | 0.045 | 1.327 | 0.285 | 1 |
|  | A | 3_vs_1 | 1 | 46.378 | 0.354 | 13.684 | 0.001 | **0.012*** |
|  | A | 2_vs_1 | 1 | 34.177 | 0.281 | 9.767 | 0.001 | **0.012*** |
|  | B | 3_vs_2 | 1 | 11.926 | 0.136 | 4.423 | 0.025 | 0.3 |
|  | B | 3_vs_1 | 1 | 36.659 | 0.296 | 11.354 | 0.001 | **0.012*** |
|  | B | 2_vs_1 | 1 | 10.804 | 0.103 | 2.883 | 0.041 | 0.492 |
|  | C | 1_vs_2 | 1 | 18.683 | 0.172 | 4.788 | 0.002 | **0.024*** |
|  | C | 1_vs_3 | 1 | 29.398 | 0.223 | 7.449 | 0.001 | **0.012*** |
|  | C | 2_vs_3 | 1 | 1.256 | 0.028 | 0.658 | 0.480 | 1 |
|  | D | 1_vs_2 | 1 | 6.915 | 0.042 | 1.975 | 0.071 | 0.852 |
|  | D | 1_vs_3 | 1 | 5.920 | 0.024 | 1.441 | 0.219 | 1 |
|  | D | 2_vs_3 | 1 | 1.431 | 0.008 | 0.333 | 0.784 | 1 |
|  | 1 | A_vs_B | 1 | 1.229 | 0.060 | 1.838 | 0.140 | 1 |
|  | 1 | A_vs_C | 1 | 3.677 | 0.118 | 3.608 | 0.061 | 1 |
|  | 1 | A_vs_D | 1 | 72.139 | 0.353 | 22.870 | 0.001 | **0.018*** |
|  | 1 | B_vs_C | 1 | 1.782 | 0.039 | 1.130 | 0.297 | 1 |
|  | 1 | B_vs_D | 1 | 72.056 | 0.326 | 20.778 | 0.001 | **0.018*** |
|  | 1 | C_vs_D | 1 | 44.704 | 0.221 | 11.657 | 0.001 | **0.018*** |
|  | 2 | A_vs_B | 1 | 9.271 | 0.183 | 6.056 | 0.001 | **0.018*** |
|  | 2 | A_vs_C | 1 | 2.116 | 0.082 | 2.143 | 0.156 | 1 |
|  | 2 | A_vs_D | 1 | 62.635 | 0.503 | 28.307 | 0.001 | **0.018*** |
|  | 2 | B_vs_C | 1 | 3.782 | 0.058 | 1.426 | 0.213 | 1 |
|  | 2 | B_vs_D | 1 | 60.956 | 0.380 | 16.583 | 0.001 | **0.018*** |
|  | 2 | C_vs_D | 1 | 40.488 | 0.332 | 11.906 | 0.001 | **0.018*** |
|  | 3 | A_vs_B | 1 | 8.001 | 0.239 | 7.214 | 0.001 | **0.018*** |
|  | 3 | A_vs_C | 1 | 9.632 | 0.137 | 3.800 | 0.005 | 0.09 |
|  | 3 | A_vs_D | 1 | 54.214 | 0.484 | 39.342 | 0.001 | **0.018*** |
|  | 3 | B_vs_C | 1 | 0.874 | 0.011 | 0.266 | 0.813 | 1 |
|  | 3 | B_vs_D | 1 | 75.024 | 0.486 | 40.722 | 0.001 | **0.018*** |
|  | 3 | C_vs_D | 1 | 80.449 | 0.413 | 30.902 | 0.001 | **0.018*** |
| **2019** |  |  |  |  |  |  |  |  |
|  | A | 3_vs_2 | 1 | 24.725 | 0.412 | 10.498 | 0.003 | **0.036*** |
|  | A | 3_vs_1 | 1 | 28.937 | 0.626 | 18.431 | 0.004 | **0.048*** |
|  | A | 2_vs_1 | 1 | 3.425 | 0.110 | 1.240 | 0.262 | 1 |
|  | B | 1_vs_2 | 1 | 22.642 | 0.242 | 7.992 | 0.001 | **0.012*** |
|  | B | 1_vs_3 | 1 | 41.216 | 0.362 | 14.194 | 0.001 | **0.012*** |
|  | B | 2_vs_3 | 1 | 20.428 | 0.199 | 7.475 | 0.004 | **0.048*** |
|  | C | 3_vs_2 | 1 | 0.782 | 0.043 | 0.401 | 0.696 | 1 |
|  | C | 3_vs_1 | 1 | 16.503 | 0.285 | 4.389 | 0.007 | 0.084 |
|  | C | 2_vs_1 | 1 | 12.540 | 0.225 | 3.493 | 0.013 | 0.156 |
|  | D | 1_vs_2 | 1 | 9.094 | 0.056 | 1.788 | 0.127 | 1 |
|  | D | 1_vs_3 | 1 | 12.024 | 0.078 | 2.628 | 0.056 | 0.672. |
|  | D | 2_vs_3 | 1 | 0.495 | 0.008 | 0.252 | 0.840 | 1 |
|  | 1 | A_vs_B | 1 | 2.478 | 0.063 | 1.547 | 0.171 | 1 |
|  | 1 | A_vs_C | 1 | 9.995 | 0.427 | 8.926 | 0.001 | **0.018*** |
|  | 1 | A_vs_D | 1 | 69.710 | 0.811 | 102.746 | 0.001 | **0.018*** |
|  | 1 | B_vs_C | 1 | 4.899 | 0.092 | 1.927 | 0.139 | 1 |
|  | 1 | B_vs_D | 1 | 84.862 | 0.624 | 51.430 | 0.001 | **0.018*** |
|  | 1 | C_vs_D | 1 | 31.575 | 0.532 | 22.762 | 0.001 | **0.018*** |
| *Continued* | | | | | | | | |

| ***Table S3.*** *Continued* | | | | | | | | |
| --- | --- | --- | --- | --- | --- | --- | --- | --- |
|  | 2 | A_vs_B | 1 | 6.600 | 0.160 | 4.177 | 0.024 | 0.432 |
|  | 2 | A_vs_C | 1 | 5.134 | 0.357 | 6.659 | 0.001 | **0.018*** |
|  | 2 | A_vs_D | 1 | 61.527 | 0.744 | 64.077 | 0.001 | **0.018*** |
|  | 2 | B_vs_C | 1 | 5.621 | 0.121 | 2.747 | 0.057 | 1 |
|  | 2 | B_vs_D | 1 | 92.868 | 0.638 | 52.760 | 0.001 | **0.018*** |
|  | 2 | C_vs_D | 1 | 28.320 | 0.509 | 20.750 | 0.001 | **0.018*** |
|  | 3 | A_vs_B | 1 | 6.350 | 0.294 | 5.404 | 0.026 | 0.468 |
|  | 3 | A_vs_C | 1 | 12.657 | 0.232 | 3.028 | 0.040 | 0.72 |
|  | 3 | A_vs_D | 1 | 22.416 | 0.467 | 15.781 | 0.001 | **0.018*** |
|  | 3 | B_vs_C | 1 | 7.898 | 0.122 | 2.364 | 0.085 | 1 |
|  | 3 | B_vs_D | 1 | 38.178 | 0.485 | 23.532 | 0.001 | **0.018*** |
|  | 3 | C_vs_D | 1 | 34.992 | 0.343 | 11.476 | 0.001 | **0.018*** |
|  | | | | | | | | |

## Table S4

| ***Table S4.*** *Mean values of abundance and dry weight per Zone and year. The range of values is provided in parentheses.* | | | | | |
| --- | --- | --- | --- | --- | --- |
|  | | **Abundance** | | **Dry weight** | |
|  |  | **ind. m^-2^** | **ind. m^-3^** | **g m^-2^** | **mg m^-3^** |
| 2017 | Zone A | 182,810 | 18,703 | 1.8 | 37.2 |
|  |  | (49,493 - 419,200) | (990 - 45,611) | (0.6 - 3.9) | (3.4 - 100.3) |
|  | Zone B | 151,765 | 11,338 | 2.2 | 37.5 |
|  |  | (58,880 - 255,680) | (2,387 - 39,335) | (0.1 - 5.5) | (3.2 - 118.9) |
|  | Zone C | 176,458 | 11,348 | 5.5 | 81.7 |
|  |  | (54,864 - 357,760) | (1,097-40,021) | (0.7 - 11.1) | (10.9 - 248.1) |
|  | Zone D | 193,305 | 12,711 | 6.1 | 95.1 |
|  |  | (27,920 -724,800) | (1,117 - 65,024) | (0.4 - 30.3) | (7.5 - 437.5) |
|  | All area | 176,959 | 13,195 | 4.2 | 67.0 |
|  |  | (27,920 - 724,800) | (990 - 65,024) | (0.1 - 30.3) | (3 - 438) |
| 2019 | Zone A | 61,950 | 3,587 | 1.3 | 16.6 |
|  |  | (44,160 - 104,480) | (911 - 11,840) | (0.6-2.1) | (4.1 - 43.1) |
|  | Zone B | 80,897 | 6,012 | 1.9 | 22.6 |
|  |  | (32,320 - 244,480) | (646 - 21,291) | (0.3 - 10.2) | (2.1 - 101.8) |
|  | Zone C | 154,308 | 8,047 | 2.7 | 22.5 |
|  |  | (27,008 - 651,264) | (540 - 21,465) | (0.1 - 8.4) | (4.2 - 69.1) |
|  | Zone D | 128,472 | 9,571 | 3.9 | 77.2 |
|  |  | (50,720 - 176,640) | (1,882 - 29,227) | (1 - 13.9) | (4.9 - 433.7) |
|  | All area | 112,060 | 7,345 | 2.6 | 41.0 |
|  | | (27,008 - 651,264) | (540 - 29,227) | (0.1 - 13.9) | (2 - 434) |

## Table S5

| ***Table S5.*** *Average abundance (ind. m^-2^) and contribution (%) from the SIMPER analysis on the copepod and diplostracan taxa responsible for 70% of similarity within the station groups identified by cluster analysis in 2017. The standard error is shown in parentheses. The green colour gradient indicates high and low contribution. Absence of taxa and negligible % contribution are* indicated as -. | | | | | | | | | | | |
| --- | --- | --- | --- | --- | --- | --- | --- | --- | --- | --- | --- |
| **Taxa** | **Average abundance** | | | | | **% Contribution** | | | | | |
|  | **G1** | **G2** | **G3** | **G4** | **G5** |  | **G1** | **G2** | **G3** | **G4** | **G5** |
| *Calanoides natalis* | 686.7 (0.58) | 512.0 (0.17) | 36480.0 (0.06) | 108.8 (2.43) | - |  | - | - | 11.5 | - | - |
| *Calocalanus pavo* | 2020.0 (0.59) | 256.0 (0.21) | 1312.0 (0.14) | 1071.6 (1.05) | 933.3 (0.15) |  | 4.0 | - | - | - | 6.3 |
| *Clausocalanus furcatus* | 8266.7 (0.29) | 1344.0 (0.12) | 4480.0 (0.09) | 2139.5 (0.78) | 1413.3 (0.07) |  | 9.2 | - | - | - | 6.3 |
| Corycaeidae | 5480.0 (0.28) | 9152.0 (0.08) | 4288.0 (0.09) | 2430.7 (0.68) | 506.7 (0.33) |  | 5.6 | 12.3 | - | - | - |
| *Mecynocera clausi* | 673.3 (0.55) | - | - | 614.0 (1.38) | 1626.7 (0.06) |  | - | - | - | - | 6.3 |
| *Oithona nana* | 446.7 (0.76) | 256.0 (0.21) | 36608.0 (0.03) | 7833.5 (0.41) | 1280.0 (0.05) |  | - | - | 7.9 | 6.7 | - |
| *Oithona plumifera* | 4740.0 (0.43) | 512.0 (0.19) | 5376.0 (0.11) | 1172.1 (1.16) | 613.3 (0.15) |  | 7.0 | - | - | - | 4.8 |
| *Oithona* spp. | 15946.7 (0.26) | 1664.0 (0.14) | 45120.0 (0.03) | 15088.4 (0.37) | 12026.7 (0.04) |  | 13.0 | - | 11.1 | 12.6 | 21.9 |
| *Oncaea curta* | 1600.0 (0.33) | 832.0 (0.12) | 36896.0 (0.03) | 20845.6 (0.24) | 1546.7 (0.09) |  | - | - | 9.4 | 10.2 | 7.6 |
| *Oncaea venusta* | 10340.0 (0.31) | 7648.0 (0.06) | 5568.0 (0.07) | 2868.8 (0.84) | 1386.7 (0.05) |  | 10.7 | 9.5 | - | 4.6 | - |
| Oncaeidae | 29746.7 (0.18) | 14592.0 (0.05) | 25984.0 (0.07) | 17223.3 (0.26) | 2773.3 (0.05) |  | 17 | 14.1 | 9.7 | 11.2 | 8.2 |
| *Paracalanus parvus** | 6666.7 (0.27) | 26976.0 (0.04) | 66656.0 (0.02) | 19645.6 (0.29) | 1973.3 (0.04) |  | 5.0 | 18.7 | 11.9 | 13.4 | - |
| *Penilia avirostris* | 1160.0 (0.41) | 4192.0 (0.07) | 320.0 (0.28) | 11870.7 (0.32) | 80.0 (0.19) |  | - | - | - | 6.1 | - |
| *Podon* spp. | 1200.0 (0.47) | - | - | 4558.1 (0.51) | 373.3 (0.09) |  | - | - | - | 5.0 | - |
| *Spinocalanus* spp. | 780 (0.89) | 192.0 (0.3) | 9056.0 (0.07) | 1096.7 (1.25) | - |  | - | - | 4.5 | - | - |
| *Subeucalanus* spp. | 2786.7 (0.4) | 11456.0 (0.04) | 24128.0 (0.03) | 491.2 (1.54) | - |  | - | 8.0 | 5.7 | - | - |
| *Temora stylifera* | 1593.3 (0.47) | 5824.0 (0.08) | 1152.0 (0.13) | 5107.0 (0.48) | 720.0 (0.19) |  | - | 8.2 | - | 4.7 | 5.6 |
| * *Paracalanus parvus* species complex | | | | | | | | | | |  |

## Table S6

| ***Table S6.*** *Average abundance (ind. m^-2^) and contribution (%) from the SIMPER analysis on the copepod and diplostracan taxa responsible for 70% of similarity within the station groups identified by cluster analysis in 2019. The standard error is shown in parentheses. The green colour gradient indicates high and low contribution. Absence of taxa and negligible % contribution are* indicated as -. | | | | | | | | | | | |
| --- | --- | --- | --- | --- | --- | --- | --- | --- | --- | --- | --- |
| **Taxa** | **Average abundance** | | | | | **Contribution** | | | | | |
|  | **G1** | **G2** | **G3** | **G4** | **G5** |  | **G1** | **G2** | **G3** | **G4** | **G5** |
| *Acartia clausi* | 23.7 (2.43) | 4998.6 (0.18) | 16640.0 (0.02) | 796.8 (0.53) | 6556.7 (0.12) |  | - | 7.9 | 6.7 | - | 18.1 |
| *Calanoides natalis* | 296.3 (0.93) | 1183.2 (0.3) | 12544.0 (0.03) | 657.6 (0.62) | 70.0 (0.62) |  | - | - | 6.6 | - | - |
| *Calanus* spp. | 1357.0 (0.68) | 5348.1 (0.13) | 4608.0 (0.04) | 2130.7 (0.35) | 263.3 (0.43) |  | - | 6.3 | - | 3.8 | - |
| *Calocalanus pavo* | 1143.7 (0.8) | 919.0 (0.38) | 6400.0 (0.02) | 2227.2 (0.51) | 33.3 (1.01) |  | - | - | - | 6.9 | - |
| *Centropages typicus* | 195.6 (1.26) | 928.8 (0.39) | 7680.0 (0.03) | 1613.9 (0.33) | 203.3 (0.52) |  | - | - | 4.9 | - | - |
| *Clausocalanus furcatus* | 3525.9 (0.53) | 7791.6 (0.17) | 15872.0 (0.01) | 2308.8 (0.5) | 1660.0 (0.18) |  | 6.0 | 10.2 | - | 6.6 | - |
| *Corycaeidae* | 5140.7 (0.48) | 1575.4 (0.33) | 6656.0 (0.03) | 2204.8 (0.55) | 173.3 (0.59) |  | 7.6 | 4.4 | - | 7.0 | - |
| *Euterpina acutifrons* | 503.7 (0.97) | 3533.1 (0.18) | - | 1023.5 (0.41) | 8520.0 (0.09) |  | - | - | - | - | 13.7 |
| *Mecynocera clausi* | 35.6 (2.81) | 183.8 (0.73) | 2560.0 (0.03) | 832.0 (0.69) | 90.0 (0.53) |  | - | - | - | 3.2 | - |
| *Oithona nana* | 432.6 (1.25) | 10607.6 (0.14) | 25088.0 (0.02) | 1872.0 (0.39) | 18893.3 (0.06) |  | - | 10.9 | 9.8 | 4.3 | 22.6 |
| *Oithona plumifera* | 3265.2 (0.56) | 1785.4 (0.29) | 7680.0 (0.02) | 1432.5 (0.63) | 753.3 (0.22) |  | 5.4 | - | - | 5.0 | - |
| *Oithona* spp. | 11271.1 (0.32) | 2349.9 (0.28) | 27136.0 (0.02) | 1997.9 (0.46) | 1923.3 (0.14) |  | 11.1 | 4.9 | 9.8 | 4.9 | - |
| *Oncaea curta* | 527.4 (0.74) | 13128.2 (0.07) | 33024.0 (0.01) | 5198.4 (0.23) | 4746.7 (0.11) |  | - | 4.5 | 7.1 | 6.3 | 8.3 |
| *Oncaea venusta* | 16568.9 (0.25) | 18441.8 (0.08) | 65280.0 (0.01) | 2346.1 (0.49) | 1380.0 (0.17) |  | 13.3 | 11.4 | 9.2 | 6.6 | - |
| Oncaeidae | 14047.4 (0.28) | 1156.9 (0.29) | 1280.0 (0.06) | 2430.9 (0.38) | 2066.7 (0.14) |  | 12.9 | - | - | 5.5 | 6.3 |
| *Paracalanus parvus** | 10554.1 (0.27) | 12665.4 (0.1) | 34816.0 (0.01) | 4216.0 (0.33) | 1023.3 (0.26) |  | 9.4 | 11.0 | 7.3 | 8.1 | 5.1 |
| *Spinocalanus* spp. | 758.5 (0.86) | 182.2 (0.73) | 2560 .0(0.03) | 928.0 (0.72) | 550.0 (0.27) |  | - | - | - | 3.2 | - |
| *Subeucalanus* spp. | 5543.7 (0.39) | 1186.5 (0.34) | 7168.0 (0.02) | 984.0 (0.57) | 193.3 (0.38) |  | 6.8 | - | - | - | - |
| *Temora longicornis* | - | 2105.4 (0.21) | 34304.0 (0.01) | 13.3 (3.09) | 66.7 (0.71) |  | - | - | 9.0 | - | - |
| **Paracalanus parvus species complex* | | | | | | | | | | | |


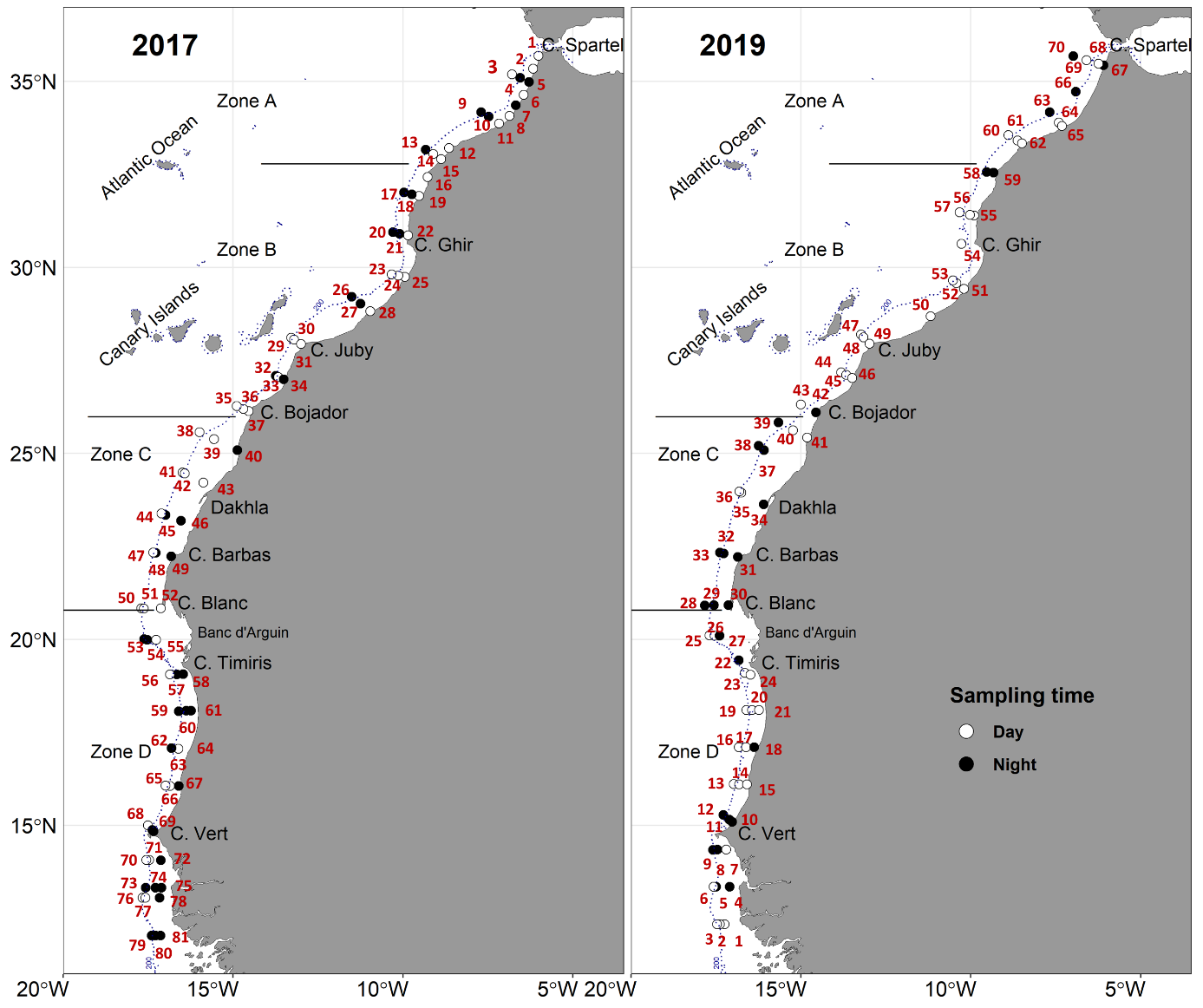


## Figure S1

Maps of the study area in 2017 and 2019. The time of sampling (Day and Night) for each station and the four upwelling Zones (A, B, C, D) along the study area are shown (2017: 44 at day and 38 at night; 2019: 45 at day and 31 at night). The number of stations is provided in red.


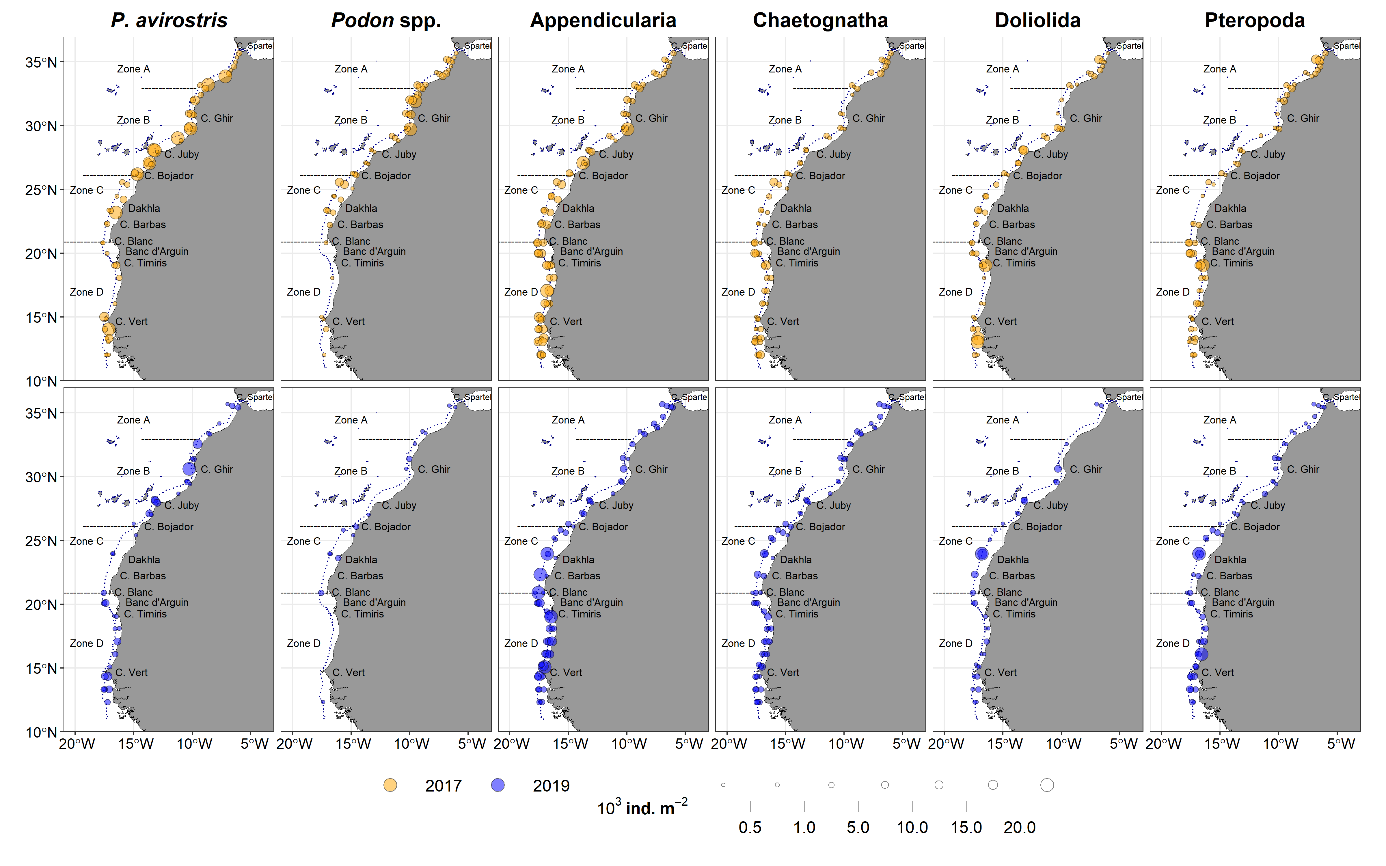


## Figure S2

Abundance (ind. m^-2^) distribution maps for the major mesozooplankton groups in 2017 and 2019.


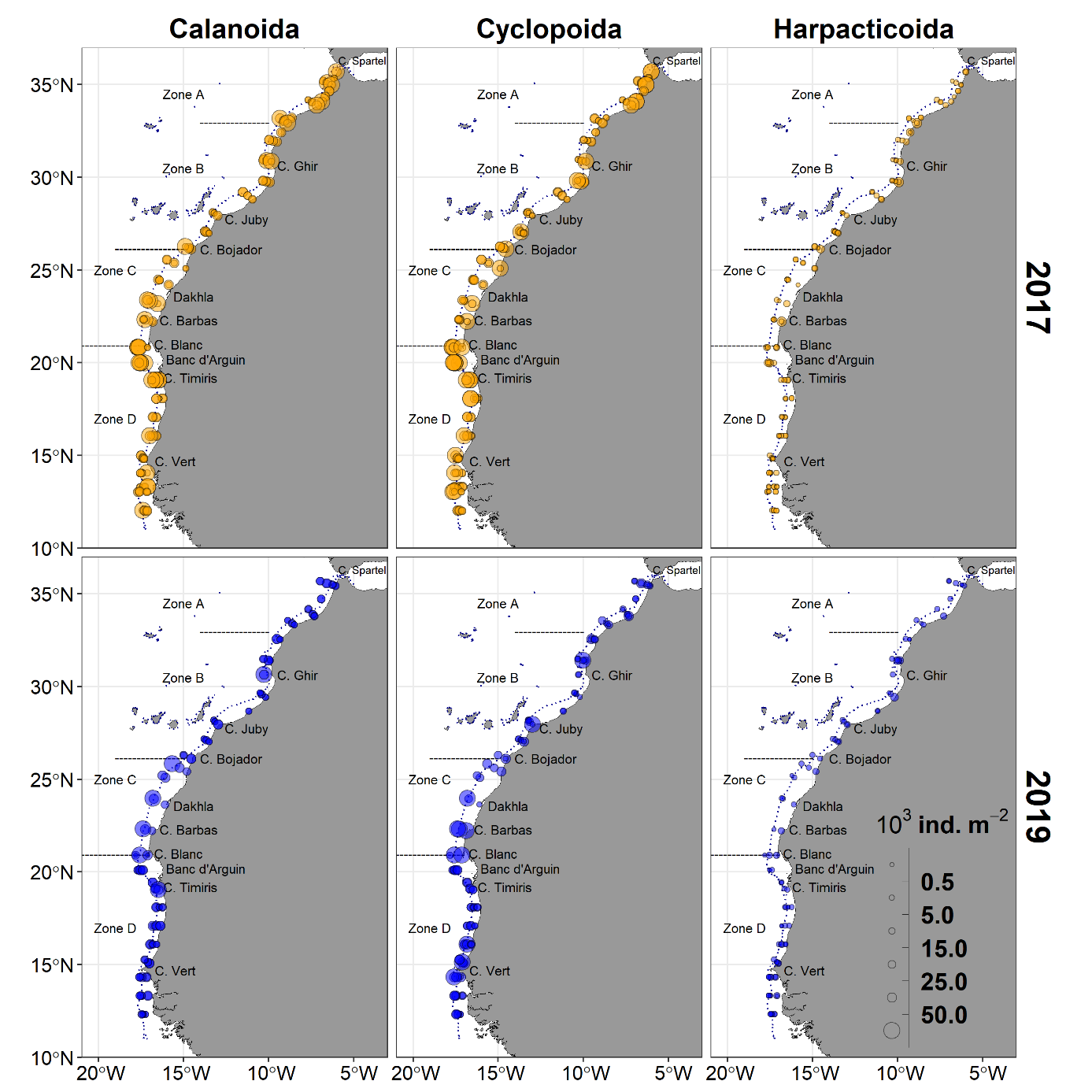


## Figure S3

Abundance distribution maps for the major copepod orders in 2017 and 2019.

##
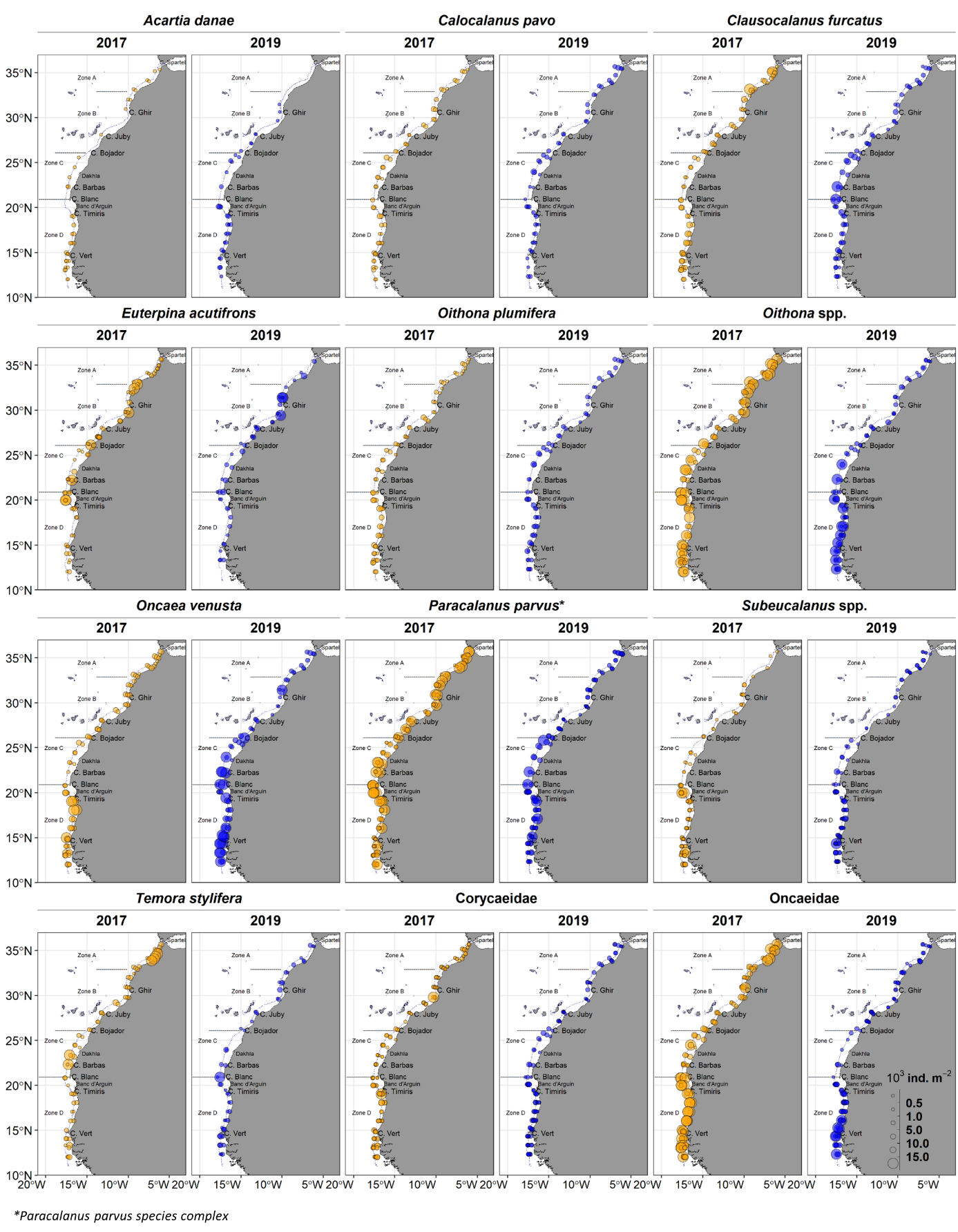
Figure S4

Abundance distribution maps of major copepod taxa broadly distributed along the study area in 2017 and 2019.

##
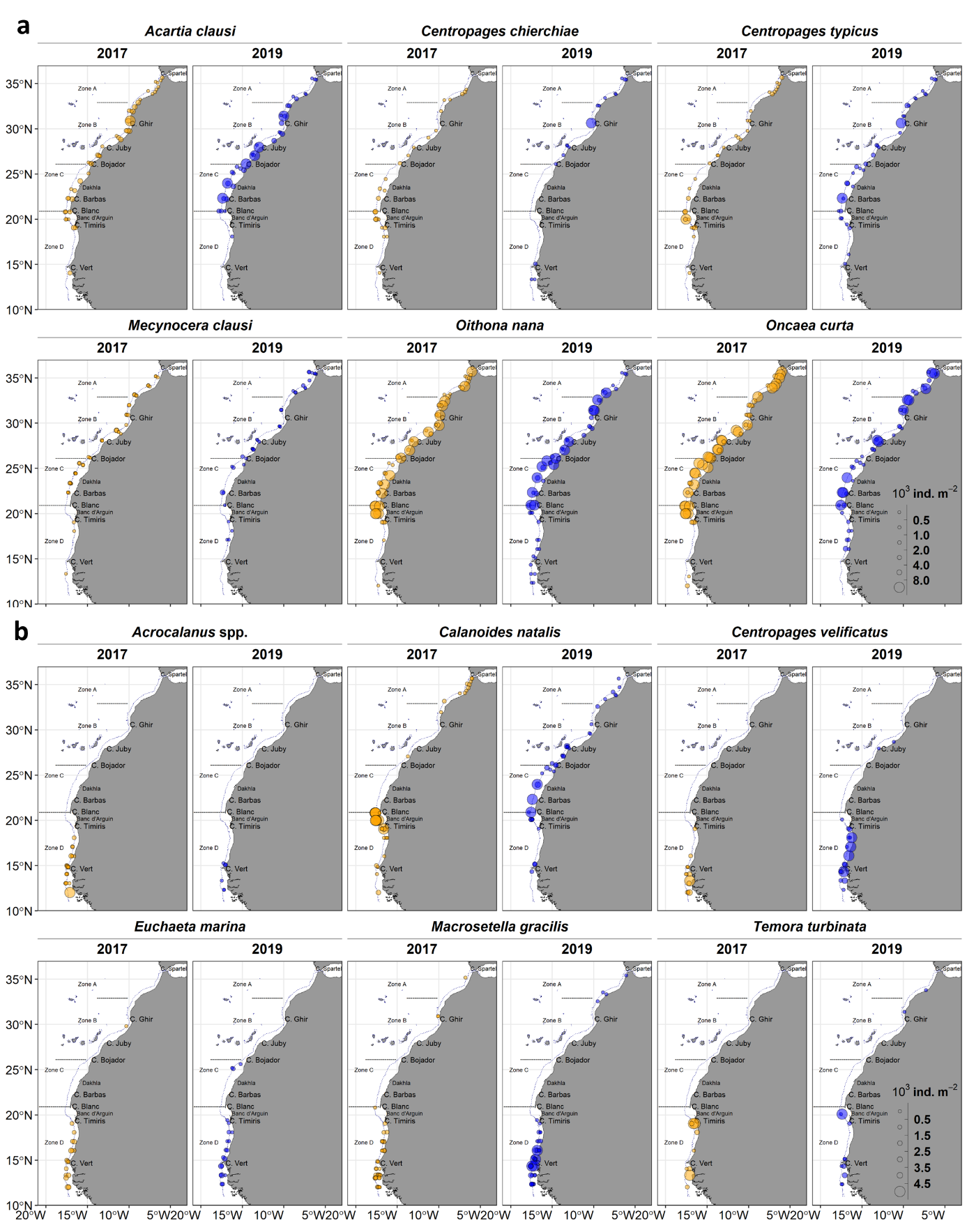
Figure S5

Abundance distribution maps of major copepod taxa distributed north (a) and south (b) of Cape Blanc in 2017 and 2019.


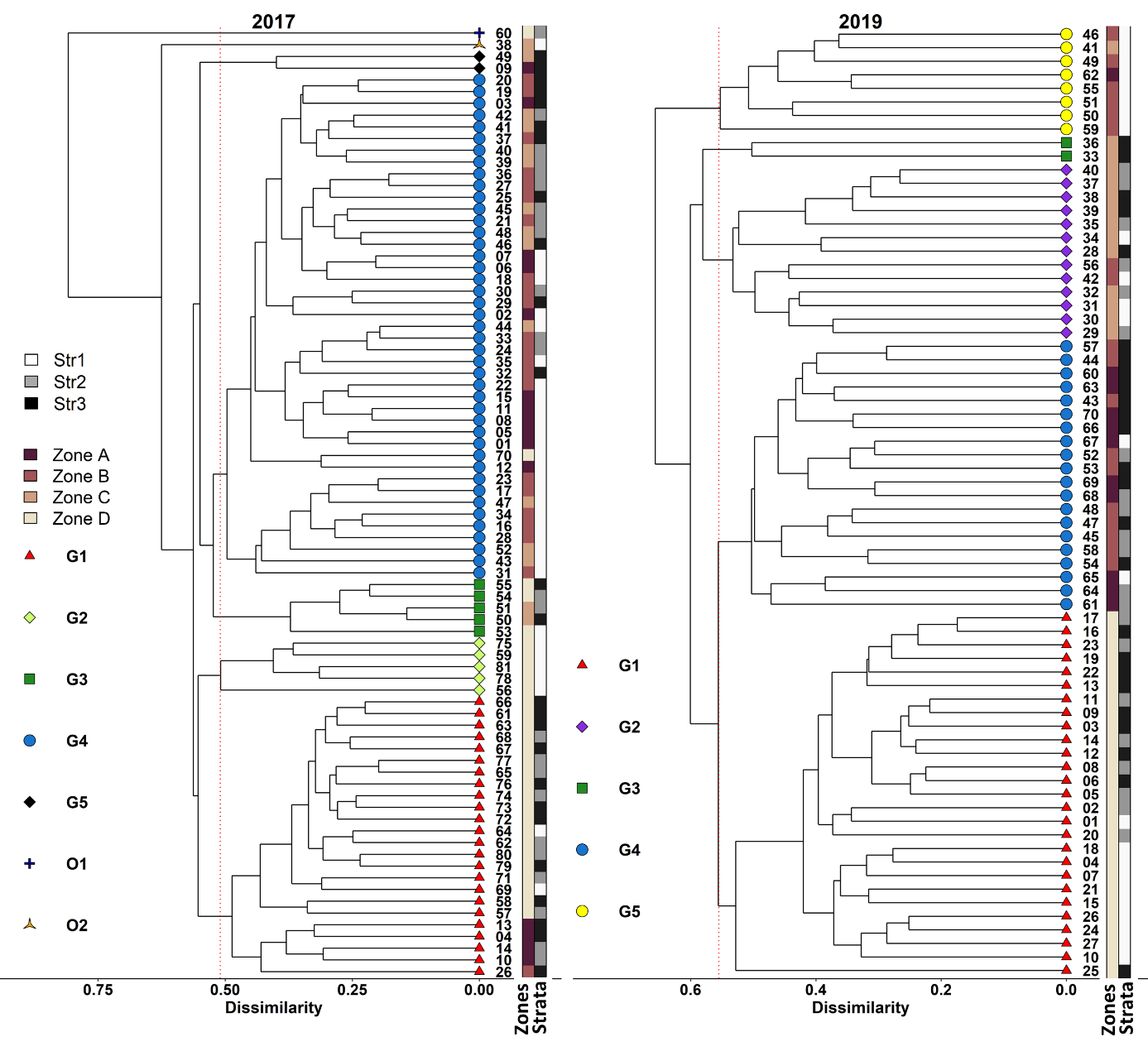


## Figure S6

Dendrograms of cluster analyses based on Bray-Curtis’s dissimilarity index and square-root transformed copepod and diplostracan abundance data, in 2017 and 2019 (outlier stations: O1, O2 due to low abundances). The coloured sidebar indicates the location of the stations according to the latitudinal upwelling Zones (A-D) and the bathymetric strata (Str1-3).

##
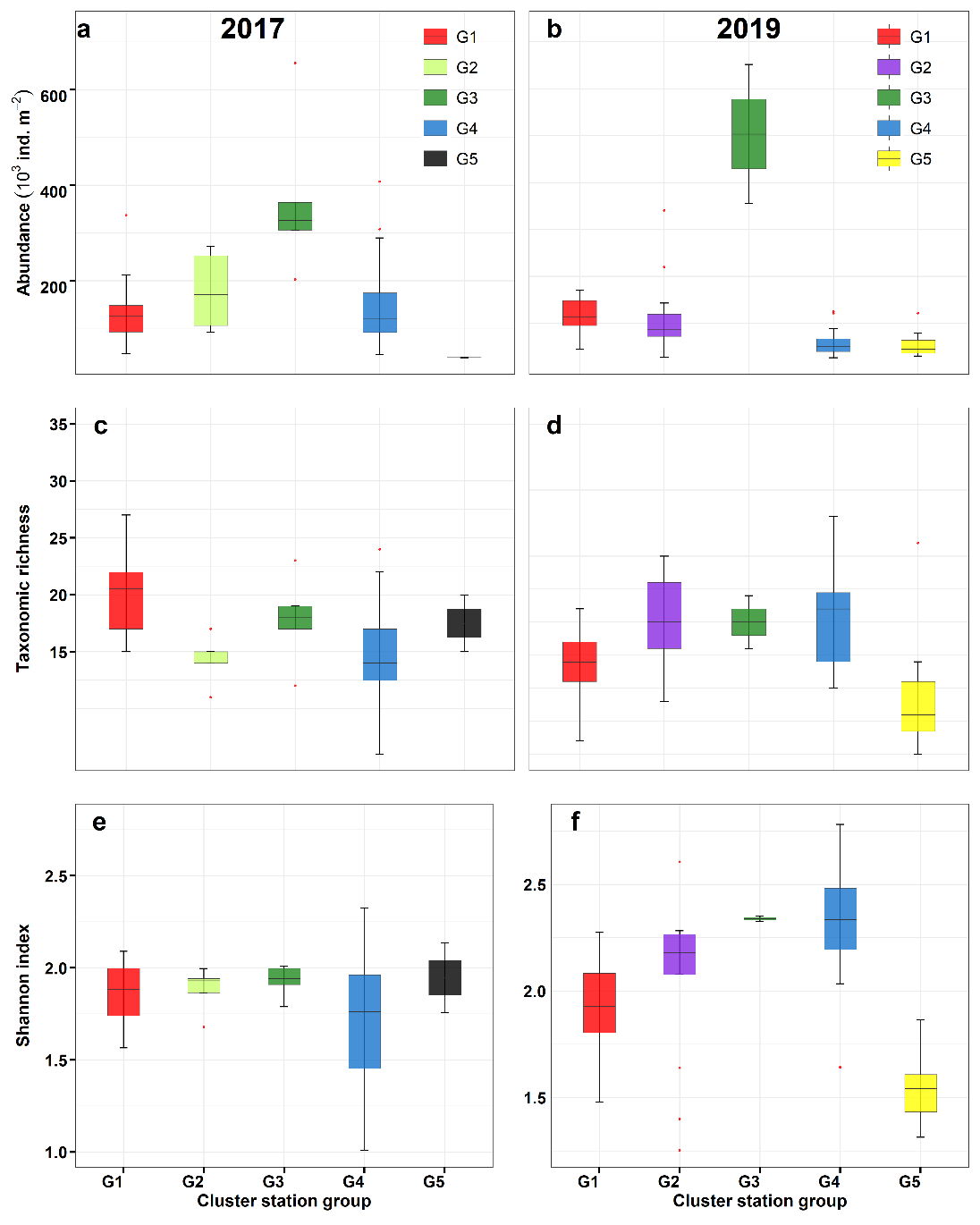
Figure S7

Box-plots showing mean abundance of the copepod sand diplostracans and the diversity indices (taxonomic richness and Shannon index) at the genus level across the cluster groups in 2017 (a, c, e) and 2019 (b, d, f). The size of the Box-Plot is determined by the upper and lower quartile, with the median indicated by a horizontal black line within the box. Outliers are represented by the red dots outside the box.
