# Supplementary material for: Spatio-temporal variability of mesozooplankton distribution along the Canary Current Large Marine Ecosystem: a regional perspective
Source: J Plankton Res. 2025 Jan 30;47(1):fbae079. doi: 10.1093/plankt/fbae079 (PMC11781819; doi:10.1093/plankt/fbae079)
Supplement: Supplementary_data_2_fbae079 [file supplementary_data_2_fbae079.docx]

# SUPPLEMENTARY DATA 2

Our protocol involved sample collection from the 0-200 m layer and should exclude the effect of diel vertical migration at stations located at the 30 m and 100 m isobaths (Strata 1 and 2, respectively). For these stations, where the entire water column was sampled the vertical migration of particular taxa would not influence the results. Therefore, we believe that this aspect should alleviate concerns regarding potential biases for the first two Strata. However, stations along the 500 m isobath or deeper (Stratum 3: 25 % of our station grid in each year) may be susceptible to the influence of diel vertical migration, which could result in increased mesozooplankton abundance and biomass at night. This migration could also impact the observed assemblage structure.

Disentangling these effects is challenging due to the lack of day and night samples (replicates) collected at the same stations. As a result, confounding effects related to station positions cannot be fully eliminated through statistical analysis using the current dataset. Therefore, any comparison between day and night samples in Stratum 3 would require assumptions that closely spaced stations (e.g., within defined spatial bins, such as latitudinal zones in this case) exhibit similar mesozooplankton behaviour. We examined our dataset for day-night sampling effects in Stratum 3, but due to unbalanced sampling between day and night within zones (Fig. A), the dataset did not permit full two-way comparisons within Stratum 3 across all zones.


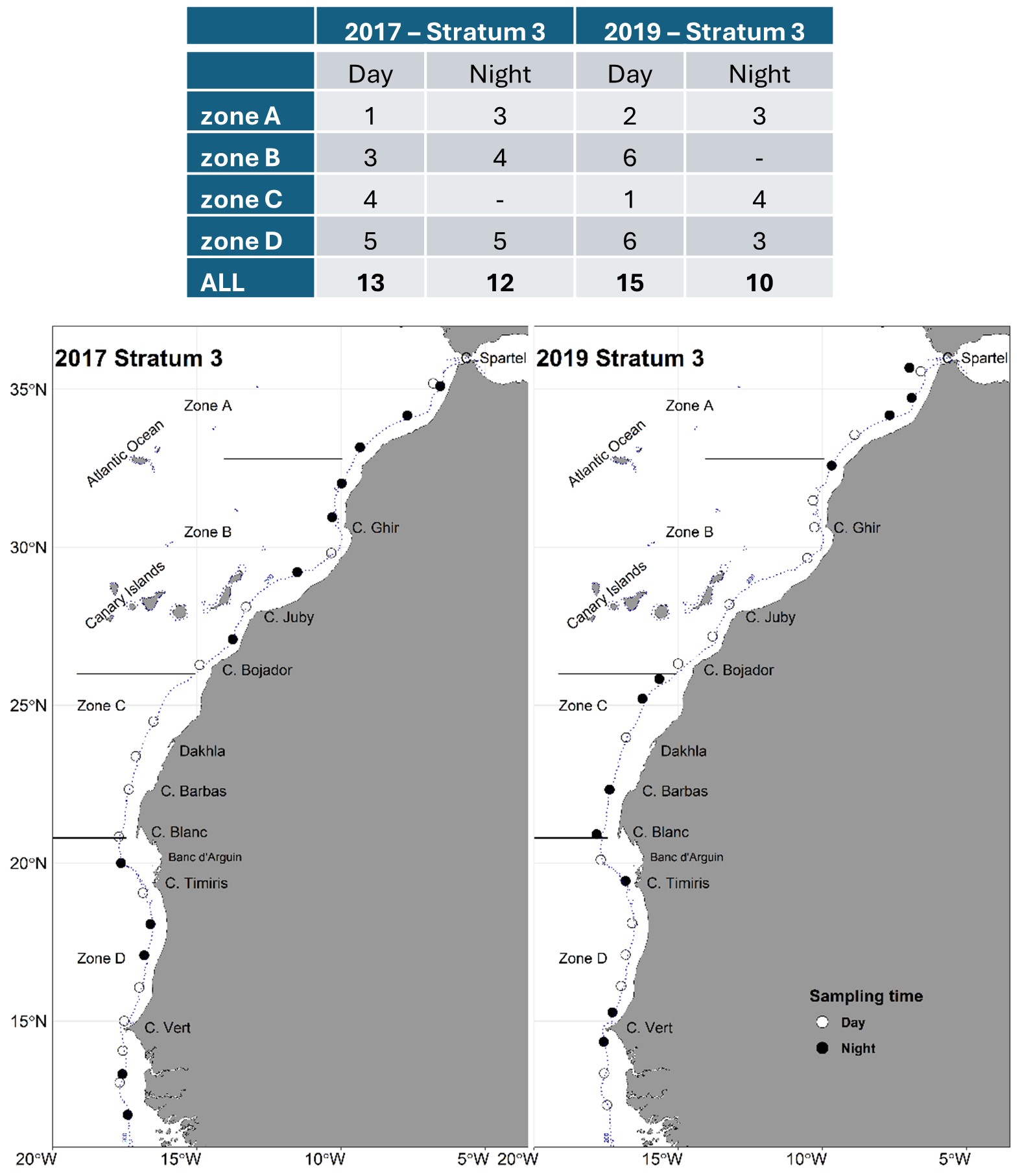


**Figure A:** Number of stations collected in Stratum 3 in the different zones (A, B, C, D) and light conditions (Day vs. Night).

This applied both to univariate data analysis (e.g., 2-way ANOVA for abundance and biomass: time vs. zone) and multivariate data analysis (e.g., PERMANOVA for assemblage structure: time vs. zone). Thus, we could only focus on the zones with >1 day or night observations within each year.

**Univariate data exploration**

For comparison, we present boxplots of total mesozooplankton abundance and dry weight for day and night sampling at stations in Stratum 3, across different zones and years (Fig. B). Focusing only on zones with similar relative contribution between daytime and nighttime samples in 2017 (i.e., zones B and D) and 2019 (i.e., zones A and D), we found no statistically significant difference between day and night samples collected in each of these zones (one-way ANOVA, Table A). Lack of statistical differences was also noted when data across Stratum 3 in the entire CCLME were pooled together. This, however, is done under the assumption that behavioral patterns regarding migration were the same across the whole CCLME region.


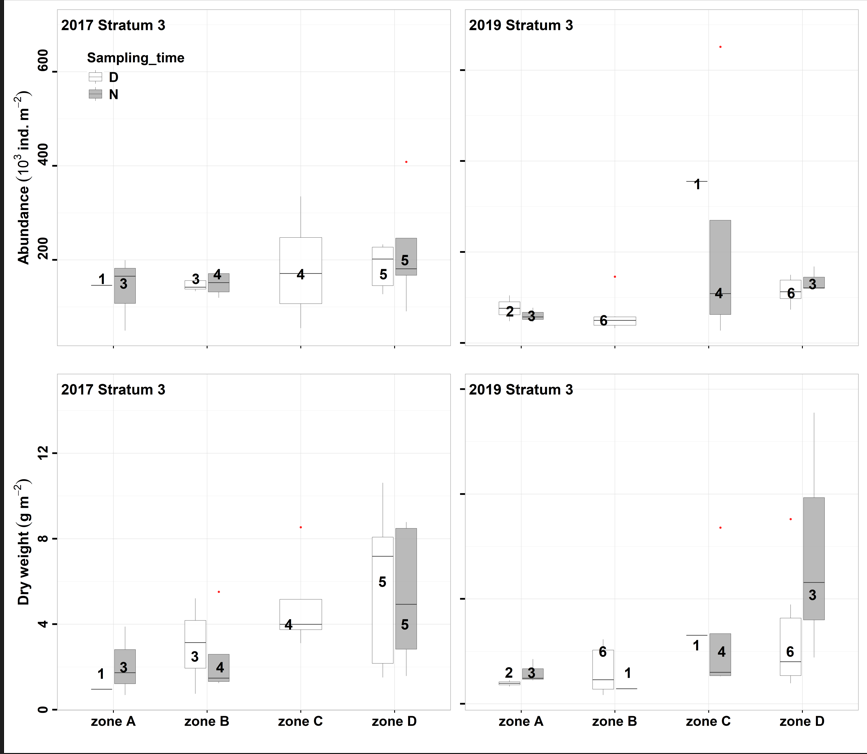


**Figure B:** Boxplots for abundance and dry-weight for each zone and each line condition. The number of stations considered in each case is provided in the box plot.

| **Table A**: Results of ANOVA analysis comparing day vs. night mean values of abundance and dry weight for each zone. | | | | | |
| --- | --- | --- | --- | --- | --- |
|  |  | **Abundance** | | **Dry weight** | |
| **Year** | **Zone** | **F** | **p** | **F** | **p** |
| 2017 | zone B | 0.004 | 0.953 | 0.061 | 0.815 |
| 2017 | zone D | 0.061 | 0.811 | 0.006 | 0.939 |
| 2019 | zone A | 0.276 | 0.636 | 1.877 | 0.264 |
| 2019 | zone D | 1.147 | 0.32 | 1.934 | 0.207 |

To further identify the effect of Stratum 3 in the boxplots of Figure 3 in our manuscript and the related statistics, we made a new plot that considered only data in Strata 1 and 2 (Fig. C c,d). We evidenced similar results in the plots and statistics regardless of the inclusion or not of Stratum 3 (Fig. C). Only in terms of biomass, zone C in 2019 was found different compared to zone D. Thus, we believe that including the samples from Stratum 3 in our dataset has not biased the comparison among zones.


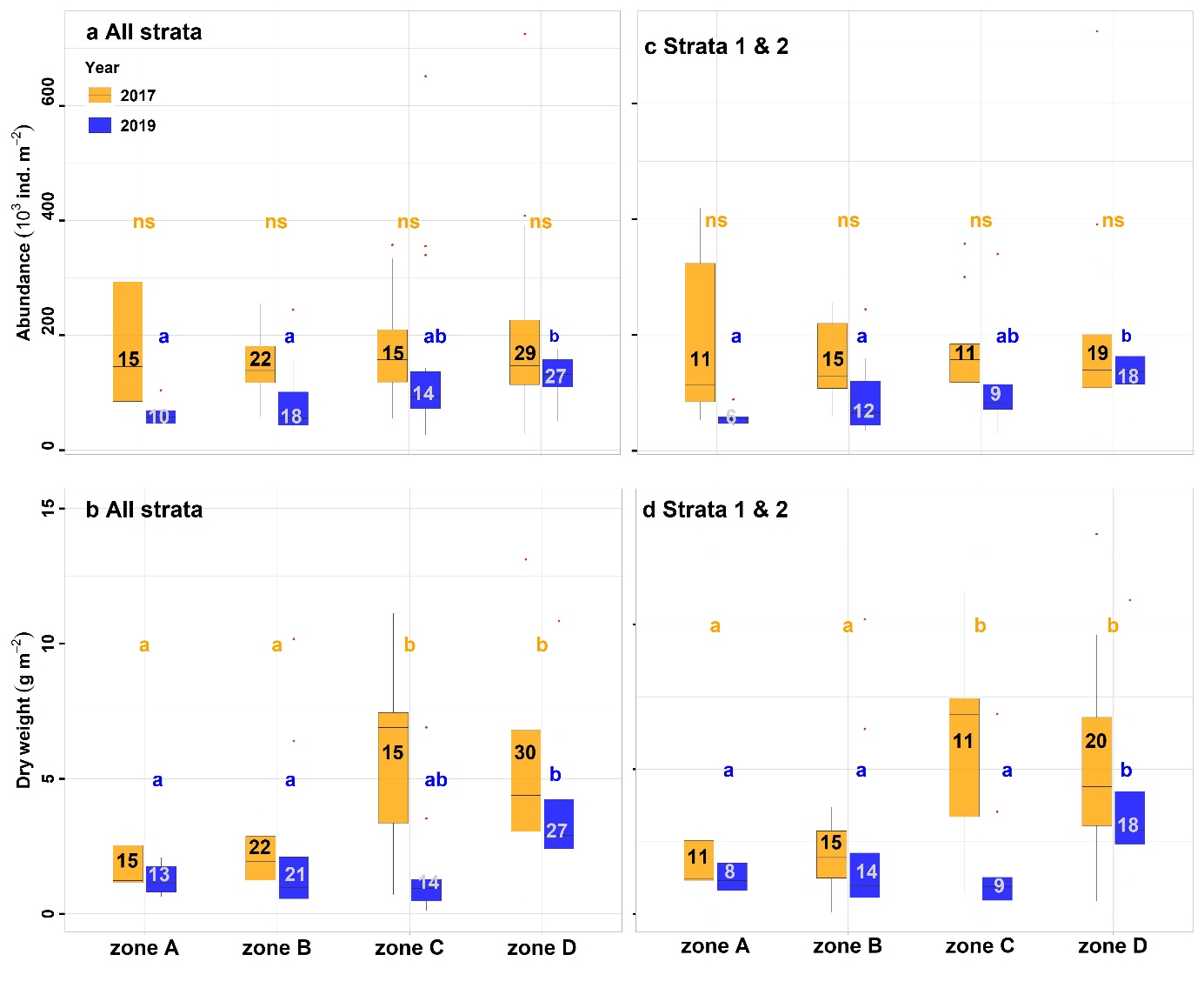


**Figure C:** Boxplots for abundance and dry-weight for each zone and each line condition. All strata considered (a, b) and only strata 1 and 2 considered (c, d)

**Multivariate data exploration in Stratum 3**

Since the dataset in Stratum 3 did not allow for a 2-way PERMANOVA (zone vs. sampling time), we could only focus on the zones with >1 day or night observations within each year (as for the univariate analysis). One-way PERMANOVA analysis between day and night samples in separate zones (zone B or zone D in 2017; zone A or zone D in 2019) showed not significant effect of the time of sampling in the assemblage structure (Table B).

| **Table B**: Results of PERMANOVA analysis comparing the copepod-cladoceran assemblage among stations sampled day vs. night for specific strata in each year | | | | | | |
| --- | --- | --- | --- | --- | --- | --- |
| **Year** | **Zone** | **df** | **Sum Of Sqs** | **R2** | **F** | **Pr(>F)** |
| **2017** | **zone B** | 1 | 0.1238 | 0.24741 | 1.6437 | 0.057 |
| **2017** | **zone D** | 1 | 0.0430 | 0.06504 | 0.5565 | 0.959 |
| **2019** | **zone A** | 1 | 0.0967 | 0.29697 | 1.2672 | 0.3 |
| **2019** | **zone D** | 1 | 0.06436 | 0.1091 | 0.8572 | 0.592 |

We also applied multivariate analyses separately on Stratum 3. The grouping of stations as identified by cluster analysis (Fig. D) and superimposed on the nMDS ordination (Fig. E), indicates that the similarity of the stations is more linked to their position and not to the time of the sampling (e.g. stations south of Cap Blanc were all grouped together regardless of the time of sampling).


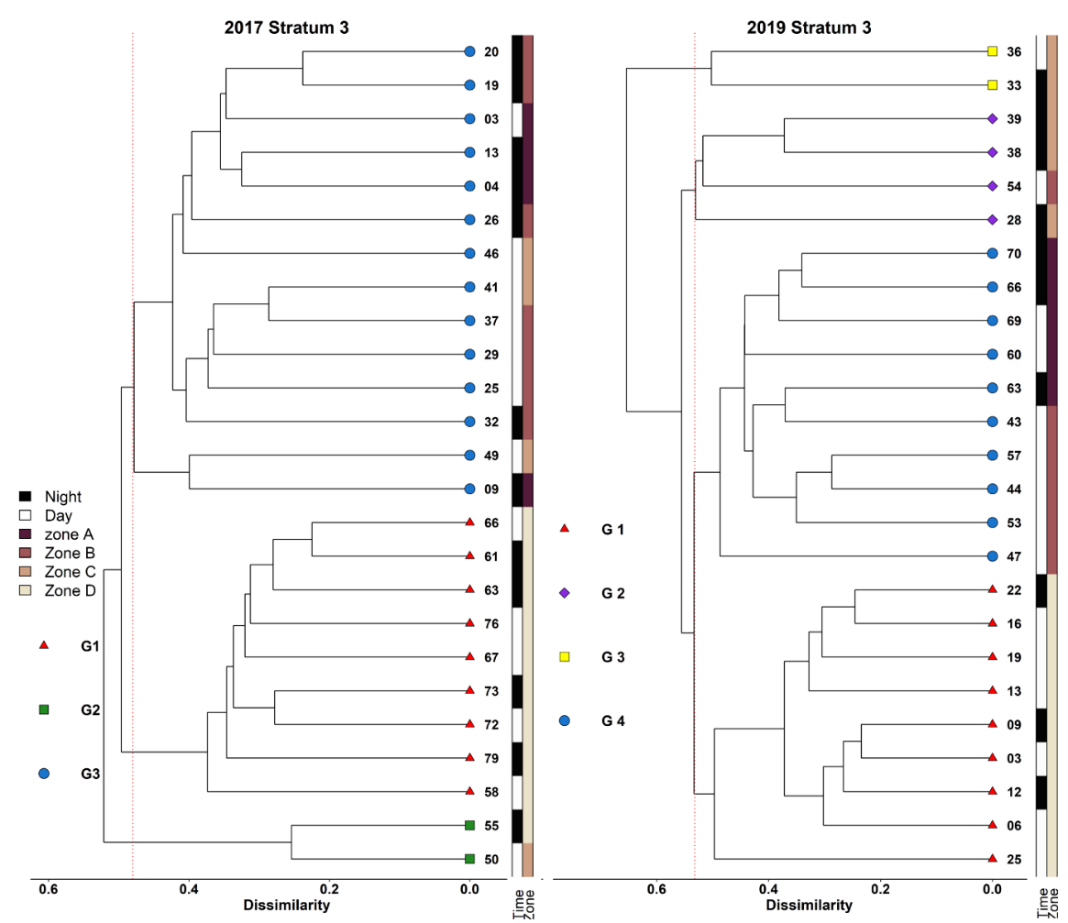


**Figure D:** Clusters of stations located at Sratum 3 based on copepod-diplostracan assemblage for the two years. Lateral bars indicate the time of sampling for each station and the zone.

Thus, we believe that any effect of the time of the day is minimal compared to the influence of hydrological properties at the sampling site. Based on these results we think it would be beneficial to include stratum 3 in the analysis together with the other 2 strata. Increasing the spatial resolution of the study strengthens the findings and makes the results more robust.

**Figure E:** nMDS ordination of samples located at Sratum 3 based on copepod-diplostracan assemblage for the two years. Day and night samples have been superimposed on the maps of the study area and the nMDS.

**Figure D:** Clusters of stations located at Sratum 3 based on copepod-diplostracan assemblage for the two years. Lateral bars indicate the time of sampling for each station and the zone.
